# A Solvent Selection Framework for Porous Organic Polymers

**DOI:** 10.1021/acs.jcim.5c02163

**Published:** 2025-11-04

**Authors:** Xue Fang, Ulzhalgas Karatayeva, John D. Worth, Merve Gumussoy Girgin, Safa Ali Al Siyabi, Dauren Mukhanov, Ella M. Gale, Charl F. J. Faul, Natalie Fey

**Affiliations:** † School of Chemistry, 1980 University of Bristol, Cantock’s Close, Bristol BS8 1TS, U.K.; ‡ Bristol Composites Institute, School of Civil, Aerospace and Mechanical Engineering, 1980 University of Bristol, University Walk, Bristol BS8 1TR, U.K.; § Department of Chemistry, Molecular Sciences Research Hub, Imperial College London, 82 Wood Lane, London W12 0BZ, U.K.

## Abstract

Selecting suitable solvents to control the morphology and properties of novel functional materials remains a significant challenge, especially when there is limited or no prior knowledge of the material and its solubility. In this work, we present a solvent selection toolkit for functional porous organic polymers. We have developed the *MLoc* algorithm for the fast determination of Hansen solubility parameters (HSPs) for novel materials. This approach requires ultraviolet and visible (UV/vis) absorbance data, measured for a number of candidate solvents using a standard laboratory setup. Based on these measurements, *MLoc* determines the HSPs for novel porous organic materials using a centroid-location algorithm based on Hansen distance. The results of this algorithm can guide the fine-tuning of both morphology and carbon-capture performance of target polymers, which we illustrate in a case study. In this example, performing the polymer synthesis in solvents with HSPs most similar to the porous material has led to CO_2_ uptake improved by 220% compared to a reported analogue (from 2.16 to 6.95 wt %). Using *MLoc*, we have also compiled a HSP database for 17 porous organic polymers, enhanced with data for over 80 reactions, sampling different conditions, which we present as a resource for future data-driven research in this area.

## Graphical abstract

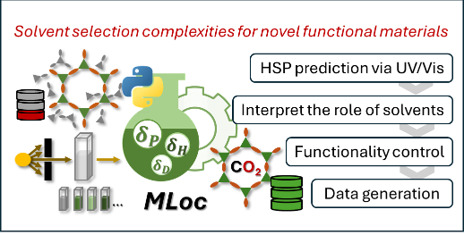

## Introduction

From reaction performance^1,2^ to purification^3^ and phase separation,^4,5^ solvents play a vital role in chemistry.^6^ Solvent selection is particularly important in industries such as pharmaceuticals and functional materials, where the associated costs of synthesis are high.^7–10^ Solvent selection can thus be critical for both efficiency and sustainability. However, selecting the “right” solvent is challenging due to limited benchmarking data and often complex solvent–solute interactions, such that this process often relies on expert knowledge.^11–14^ Additionally, the solvent selection process is usually multiobjective, where some of the target objectives, such as price, sustainability and the outcome of the synthesis (yield, selectivity, properties), may be conflicting.

While significant efforts have been made to develop quantitative solvent selection approaches,^15–25^ existing approaches rely on either large data sets or reliable details of the molecular structure of solutes. These approaches require trade-offs between accuracy, simplicity, and interpretability (see Sections S.1.1, S.1.2 in the Supporting Information). To support and inform a wide range of experiments, an ideal solvent selection approach should make scientifically sensible predictions with minimal reliance on calculations, experiments or database searches. The key challenge is to numerically describe “solvent”, a discrete variable, at an appropriate level of complexity that captures the necessary details without sacrificing interpretability or universality.^26^ Solubility parameters,^27^ based on bulk liquid properties associated with solvation energy,^15,27–30^ offer a key metric for a solvent’s ability to dissolve substances. Such solubility parameters can connect experimental observables, such as reaction rates or ultraviolet and visible (UV/vis) absorption, to the solvent’s physiochemical properties (see S.1.2 and S.1.4 in the SI for further details).^27^

In this work, we explore these challenges for conjugated microporous polymers (CMPs),^31,32^ a subclass of porous organic polymers (POPs). First reported by Cooper *et al.* in 2007, CMPs are characterized by their conjugated framework structures and microporosity;^32^ they have shown promise in carbon capture and conversion,^5,33–36^ but their performance has been found to be highly sensitive to the solvent used during synthesis.^28,37^ POPs are typically amorphous, appearing as polydisperse mixtures that form rapidly during synthesis. This presents considerable challenges for determining their chemical formulas, making bottom-up solubility prediction based on monomers barely applicable, as the polymer sequence and extent of branching are usually not known. In such cases, sample preparation and measurements can also be slow and there remains a dearth of data relating solvents to experimentally-measurable material properties for POPs. Depending on the specific chemistry, materials and methods used, many reported procedures for the synthesis and characterization of POPs take between a few hours to days.^38^ This is especially true for gas sorption measurements, which require between 1 day and 1 week per measurement per sample to complete,^39^ limiting the number of fully characterized materials. Results can be altered by catalyst loadings and stoichiometric ratios of monomers used, further complicating the collation of databases in this field. Existing approaches to the determination of POP solubility parameters, which rely on extensive screening or solute property databases, are therefore impractical.

Aiming to address the general complexities of solvent selection for novel materials, in this work we introduce a solvent selection toolkit, *MLoc*. This toolkit provides a user-friendly workflow suitable for nonspecialists, offering a novel optimization algorithm for determining HSPs from limited experimental measurements. *MLoc* operates with minimal reliance on prior knowledge of target materials and has comparatively low costs in both computational time and experimental effort, making it suitable for novel systems. In the case study presented, we demonstrate how *MLoc* can be used to improve the carbon capture capabilities of a POP material through the design of a suitable solvent system for synthesis. We also report the first Hansen solubility parameter database for POPs generated by *MLoc* to address the current data gap in this field.

## Background—Solubility Theory for Porous Organic Polymers

Solubility theories are often based on the “like dissolves like” principle. In Hildebrand and Hansen solubility theories, the affinity between two substances is quantified by their distance in the corresponding solubility space.^28,37,40^ Hildebrand solubility parameters (δ_T_ MPa^1/2^) represent the total solvent–polymer interaction.^40^ Solvent quality is described through the absolute difference between δ_T_ of two substances, Δδ_T_ (eq 1). A lower Δδ_T_ between solute (1) and solvent (2) indicates better compatibility.^40^

$$\Delta \delta_{T}=|\delta_{T}^{\left(1\right)}-\delta_{T}^{\left(2\right)}|$$ 1

Hansen solubility parameters (HSPs) further deconvolute the total interaction into dispersion (δ_D_), dipolar (δ_P_) and hydrogen-bonding interactions (δ_H_) (eq 2).^28,37^ Individually, δ_D_, δ_P_, δ_H_ have been referred to as “partial HSPs*”*. The unit of each parameter is MPa^1/2^. (See S.1.3 for further discussion.)

$$\delta_{T}^{2}=\delta_{D}^{2}+\delta_{P}^{2}+\delta_{H}^{2}$$ 2

The overall compatibility between two substances is quantified by the Hansen distance, *R*, as defined by eq 3. This concept is analogous to Δδ_T_ in that a lower *R* value indicates better compatibility. Each partial HSP can be compared individually, yielding differences (Δδ_D_, Δδ_P_ and Δδ_H_) between two substances.^28,37^ This deconvolution facilitates deeper understanding of the solvent–solute interactions and supports bottom-up experimental design. Comparing partial HSPs provides an opportunity to precisely control solvent behavior in a wide range of applications through targeted manipulation of specific interactions (dispersion, dipolar and hydrogen-bonding).^41–47^

$$R=\sqrt{4(\delta_{D}^{\left(1\right)}-\delta_{D}^{\left(2\right)}{)}^{2}+(\delta_{P}^{\left(1\right)}-\delta_{P}^{\left(2\right)}{)}^{2}+(\delta_{H}^{\left(1\right)}-\delta_{H}^{\left(2\right)}{)}^{2}}$$ 3

The Hansen distance is commonly compared with the radius of a Hansen sphere, which is a solute-specific threshold to distinguish between good and poor solvents.^48^ However, determining the Hansen sphere is nontrivial, typically relying on commercial software such as *HSPiP,* which uses binary solubility indicators.^49^ Classifying solubility, a continuous variable, into a binary ‘soluble’ or ‘insoluble’ score can introduce ambiguity and potentially compromise the fidelity and accuracy of predictions. Therefore, developing an HSP prediction algorithm based solely on the Hansen distance, independent from Hansen sphere generation, is highly desirable. To effectively apply these affinity representations, it is essential to know the solubility parameters of the solute.

Quantitative solvent selection for POPs was initiated in 2019 using Hildebrand and Hansen solubility parameters that are widely used in modern polymer science (S.1.3).^40^ This approach, namely the Bristol–Xi’an Jiaotong (BXJ) approach, is a facile strategy for fine-tuning the carbon capture performance of POPs by varying solvent conditions.^34,35^ Molecular dynamics studies have theoretically validated this approach for artificial POP synthesis.^5,33^

The BXJ approach predicts δ_T_ of POPs via a statistical method, utilizing UV/vis absorbance to represent the solubility of polymer suspensions (S.1.4, S.1.5). Explicit chemical formulas of the solute molecules are not required; instead, mixtures of polymers can simply be treated as a statistically averaged ensemble. However, extending this statistical method from δ_T_ to HSPs prediction creates new challenges (S.1.6). When expanding the dimensionality of the solubility space from 1D to 3D, a single δ_T_ can correspond to multiple combinations of HSPs (Scheme 1), meaning that being solely similar in δ_*T*_ is insufficient to represent the similarity in each individual molecular interaction.^48^ This transformation creates a 3D, hence multiobjective, optimization scenario that requires advanced methods to locate the optimum. This work seeks to address this challenge by introducing the *MLoc* algorithm.

**Scheme 1:**
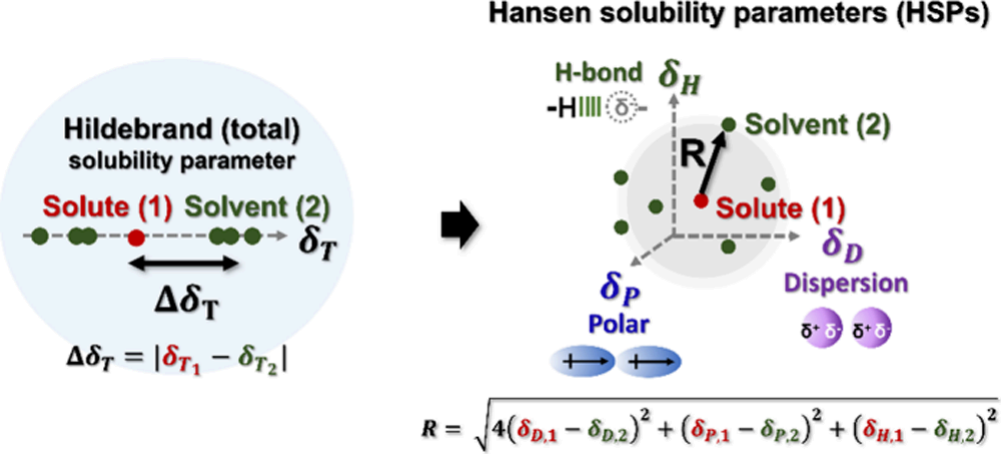
Illustration of the Deconvolution from the Hildebrand Solubility Parameter and Hansen Solubility Parameters (HSPs)^a^

a The solubility space expands from 1D to 3D.

## Results and Discussion

### Methodology—Development of *MLoc* Algorithm

*MLoc* is an optimization algorithm designed for HSP prediction of novel polymeric materials, which seeks to give interpretable results. The goal is to determine where the target material (*M*) appears in 3D Hansen space, leveraging a small set of UV/vis absorbance values measured in solvents that are well distributed in the Hansen space as the solubility score. Two key hypotheses of *MLoc* are that better solvents would (1) have a shorter Hansen distance to the target material in the Hansen space, and (2) yield a stronger UV/vis absorbance signal measured for the same aliquots of solutes.

Locating HSPs of the target material in 3D Hansen space resembles a centroid location task for a given cluster of data points. This inspired the design of *MLoc*’s core calculation architecture, building on the *k*-means cluster method.^50,51^ A *k*-means algorithm typically starts with an initial guess of the location of *k* centroids. Each centroid (*m*_*i*_) represents the mean of the corresponding cluster (*S*_*i*_). All data points are then assigned to the nearest cluster based on the Euclidean distance between each point (*x*_p_) and the centroid (*m*_*i*_) (the Assignment step), followed by creating a new series of centroids (the Update step). This “Assignment–Update” cycle is repeated until all centroids are fixed, suggesting convergence of the algorithm.^51^

Compared to a k-means algorithm, *MLoc* handles a “one-cluster” situation, where only one centroid needs to be confirmed using a given cluster of test solvents (Scheme 2). The location of the centroid corresponds to the HSP parameters for material *M*, δ_*M*_(δ_D_^(*M*)^, δ_P_^(*M*)^, δ_H_^(*M*)^).

**Scheme 2:**
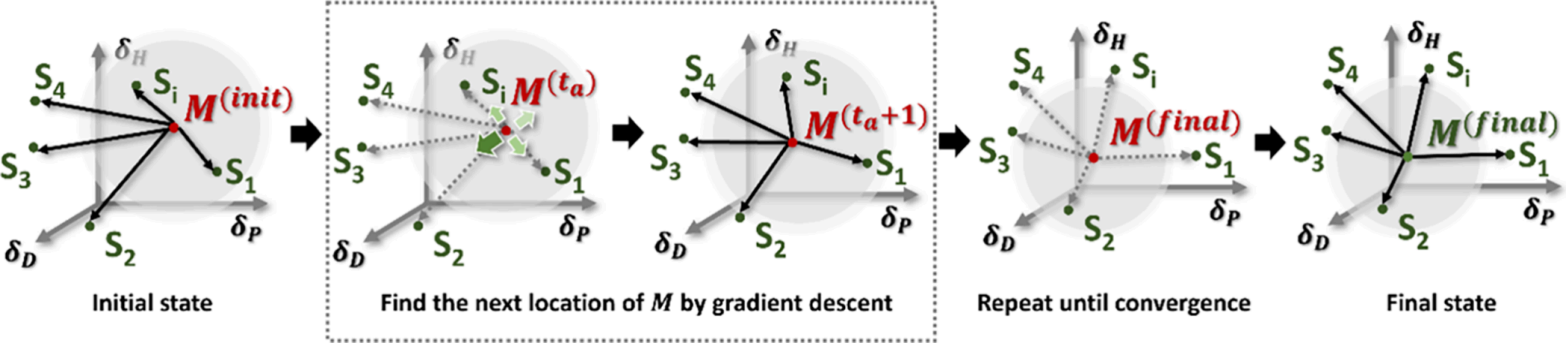
Illustration of the *MLoc* Algorithm

While such a “one-cluster” scenario is similar to taking a geometric average of HSPs of the given solvent cluster, generating a physically meaningful centroid is challenging due to the distortion of Hansen space in the dispersion dimension and the multiobjective nature of this problem (see SI Section 1.8). Two objectives are optimized simultaneously when locating the centroid using *MLoc*, namely to keep the material as close to a good solvent as possible in the Hansen space, and for the UV/vis absorbance of the dispersed material in the best solvent to be as high as possible. To account for both criteria, a simple weighted average is challenging to formulate analytically, hence requiring advanced optimization methods as developed here.

Due to the difference between Hansen distance and Euclidean distance used in a k-means algorithm, *MLoc* adopts the Hansen distance (eq 3) between a target material and a solvent as the candidate data point for the centroid. Each Hansen distance is weighted (*w*_*i*_) by the UV/vis absorbance of the saturated target material (*M*) in the corresponding solvent. Better solvents would simultaneously have a higher weight (*w*_*i*_) and lower Hansen distance (*R*_*i*_). By embedding the Beer–Lambert law and the solvent–solute compatibility rules of Hansen’s theory (eq 1and eq 3) into its optimization function here, this design enhances the theoretical robustness as well as interpretability of *MLoc.*

The location of the target material in this Hansen space is then determined by minimizing the total weighted distance between material *M* and the solvents tested, according to eq 4. The optimal combination of HSPs, δ*_M_*, is where the total weighted distance reaches the minimum.

$$\begin{array}{c} \arg\min\\ \delta_{M} \end{array}\sum_{i=1}^{n}w_{i}R_{i}(\delta_{M})$$ 4

where *R*_*i*_ is a function of δ*_M_*, *i* is the index of solvent candidates, *w*_*i*_ is the solubility weight score obtained by the UV/vis absorbance of saturated polymer suspensions, and *n* is the total number of candidates.

Equation 4 is optimized by using a gradient-descent approach[Fn fn1] (Step 3 in Figure 1)^52^ from an initial condition generated viaeq [Disp-formula eq7] (Step 2 in Figure 1). Convergence is reached when the difference of δ*_M_* between two consecutive steps (eq 5) is lower than an error threshold (default as 0.005 MPa^1/2^). The error threshold and the learning rate for gradient descent (α, default as 0.01) can both be set by users where necessary (see S.2.5).

$$\Delta =-\alpha \frac{d\sum_{i=1}^{n}w_{i}R_{i}(\delta_{M})}{d\delta_{M}}$$ 5

**Figure 1:**
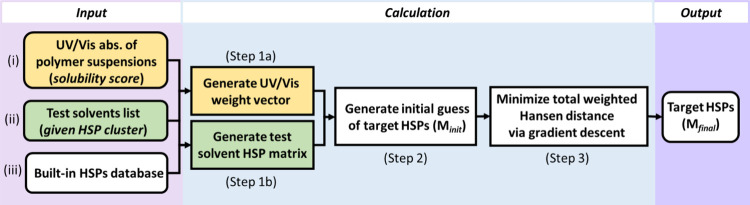
Flowchart of the MLoc optimization algorithm used to estimate HSPs of the target material. Input (i): UV/vis absorbance of polymer suspensions, serving as the solubility score; Input (ii): test solvent list, forming the HSP cluster (*i.e.*, the test solvents for UV/vis measurements); Input (iii): built-in HSPs database of solvent information, used for construction of HSP matrix. Calculation Step 1a: generating UV/vis weight vector based on Input (i) and Input (ii); Calculation Step 1b: generating test solvent HSP matrix by fetching information on solvents listed in Input (ii) from the HSP database (Input (iii); Calculation Step 2: calculating initial guess; Calculation Step 3: approaching target HSPs by minimizing the total weighted Hansen distance via gradient descent.

Instead of using a random guess at the start of this optimization, in Step 2 we utilize numerical strategies to accelerate the optimization of eq 4. The initial condition, δ_*M*_init__ (δ_D_^(*M*_init_)^, δ_P_^(*M*_init_)^, δ_H_^(*M*_init_)^), is analytically confirmed by the cost function in eq 6, which takes the squared form of the original optimization function (eq 4).

$$\begin{array}{c} \arg\min\\ \delta_{M} \end{array}\sum_{i=1}^{n}w_{i}^{2}R_{i}^{2}(\delta_{M})$$ 6

Replacing *R*_*i*_ with *R*_*i*_^2^ allows the first-order derivative associated with δ_*M*_ to be expressed exclusively by solvent HSP (δ_S_), whereas the weight term *w*_*i*_ is independent from δ_*M*_; the squared weight (*w*_*i*_^2^) does not affect the order of δ_*M*_. The result, δ_*M*_init__, corresponds to the δ_*M*_ when the first order derivative of eq 6 equals 0 MPa^1/2^ (eq 7).

$$\delta_{M_{\mathrm{init}}}=\frac{\sum_{i}w_{i}^{2}{\delta_{S}}_{i}}{\sum_{i}w_{i}^{2}}$$ 7

This approach not only reduces computing costs but also ensures that the solvents suggested are confined in an accessible solvent space. This was confirmed by conducting a grid search that randomly started the optimization with 1000 positions uniformly distributed in the Hansen space (see Section S1.7) for comparison. While *MLoc* effectively found the global minimum for each HSP within the practical solvent selection region in only 254 iteration steps, the random guess method, although achieving similar accuracy, required between 160 to 1800 steps to converge and failed in six attempts after reaching the upper limit (10,000 steps). Using a random guess approach was also more likely to predict hypothetical and impractical solvent environments. For example, a combination of δ_H_ = 0 MPa^1/2^ and moderately high δ_P_ (at ca. 13 MPa^1/2^) that does not match any real solvents was successfully avoided by *MLoc* because the prediction region was confined to physically meaningful solvent candidates.

### Operation and Outputs of *MLoc*

The workflow of the *MLoc* algorithm is shown in Figure 1. The user input consists of the UV/vis maximum absorbance value (abs.) of polymer suspensions in *n* solvent candidates (Input (i) and (ii)). The UV/vis maximum absorbance value serves as a *solubility score* for each candidate, generating a weight vector for all test solvents (Step 1a). A default candidate list for test solvents is provided by *MLoc* to maximize the diversity of candidate solvents in Hansen space and hence avoid biased sampling. These candidates are broadly distributed in each HSP dimension, with δ_D_ ranging from 14 to 19 MPa^1/2^, δ_P_ from 0 to 18 MPa^1/2^ and δ_H_ from 0 to 42 MPa^1/2^ (see Table S.2 in Section S.2.2). Test solvents are used to define a given HSP cluster for UV/vis measurements and downstream HSP optimization (Step 1b). Solvents are indexed by the corresponding Chemical Abstracts Service (CAS) numbers that can be queried from the *MLoc* built-in database (Input (iii)) to fetch their HSPs (see Section S.2.1).

The software and code development section below provides a brief overview of dependencies, while the experimental method section highlights experimental requirements. Details of experimental measurements, as well as how to install and run the algorithm, have been included in the ESI.

Once the *MLoc* calculation has been completed successfully, a summary of the results and a plot of the Hansen space (Figure 2) are saved in a spreadsheet (see S.2.8). The output plot (Figure 2) provides an overview of the relative positions of polymer and tested solvents in Hansen space. The star symbol represents the target HSPs. Plot details can be further modified using commercial software or by directly editing the source code (see S.2.7).

**Figure 2:**
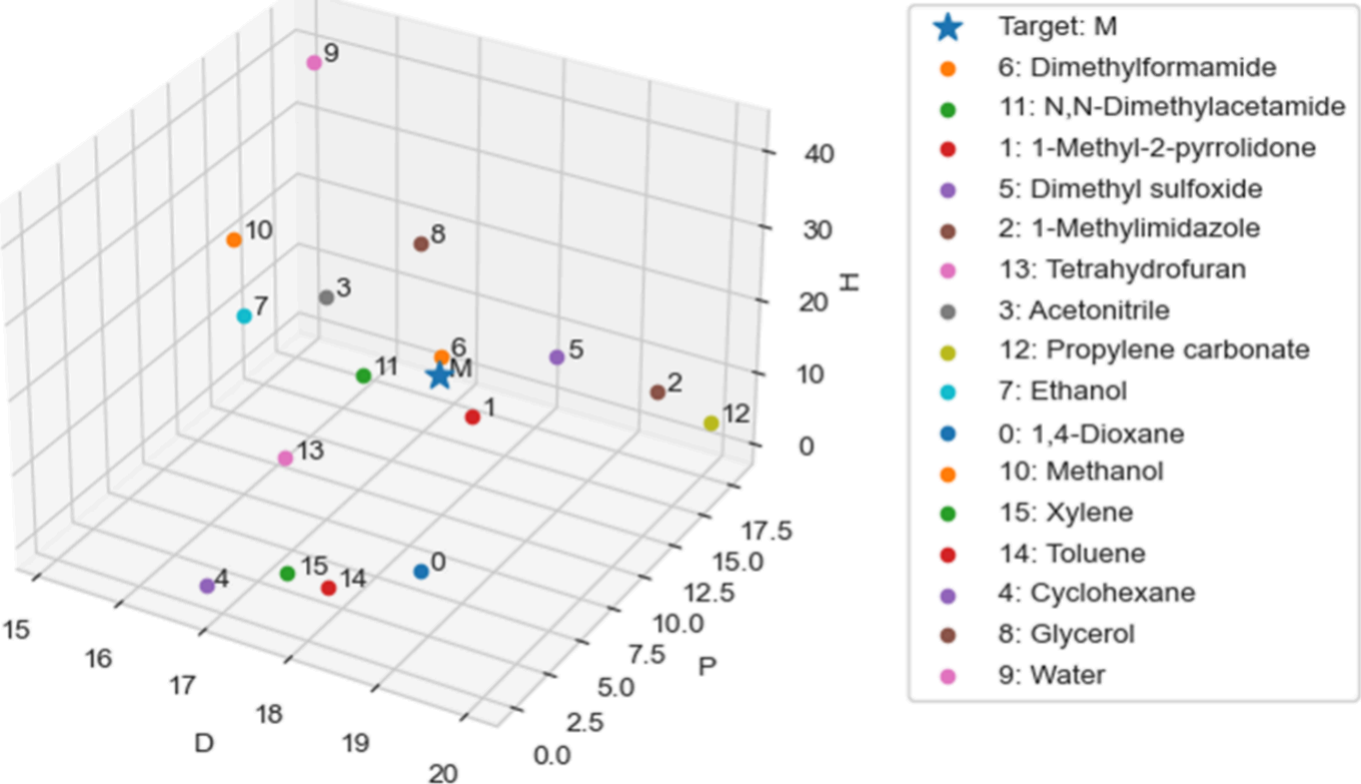
Example plot of all tested solvents and polymer of interest (*M*, denoted by the star symbol) in the 3D Hansen space.

To demonstrate this workflow, we have applied *MLoc* to predict HSPs of a novel porous organic material. This prediction enables the quantification of solvent behavior for amorphous POPs, for which HSPs are not trivial to determine. It also unlocks the use of HSP theory for optimizing and understanding solvent effects in tuning POPs functionality, such as CO_2_ uptake. The results are discussed in the Case Study section below.

#### Case Study: Application of *MLoc* in HSP Prediction and Utilization

Previous studies have shown that a smaller difference in the Hildebrand solubility parameter between the solvent and the material can result in a larger specific surface area.^34,35^ Good solubility is also expected to enhance pore volume and pore size distribution, which subsequently can facilitate CO_2_ capture.^53^ This suggests that solvents with a smaller Hansen distance (eq 3), which is conceptually similar to the difference in Hildebrand solubility parameter (eq 1), may be better suited to controlling porosity and achieving higher CO_2_ adsorption. We explored this hypothesis by applying the *MLoc* workflow and algorithm to a new carboxylic acid functionalized poly­(triphenylamine) (PTPA)-based POP, **PTPA163** (Scheme 3),^54^ synthesized in a range of different solvents. The Experimental Methods section below gives brief details, with full information included in the SI (Section 3). The key challenge for this novel material is that the solvent behavior cannot be quantified as the solubility parameters are not known.

**Scheme 3:**
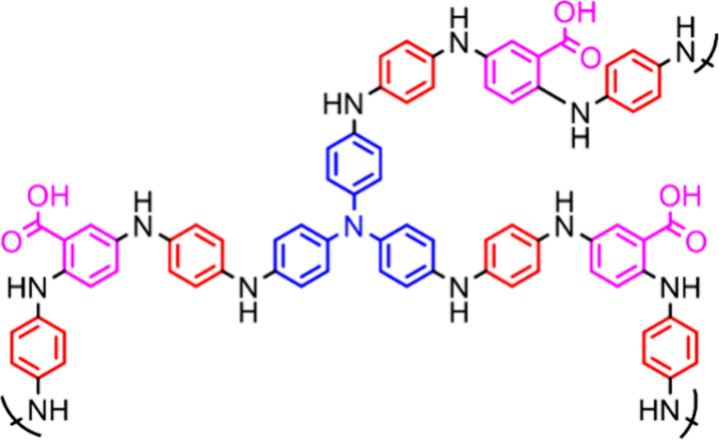
Chemical Structure of PTPA163^a^

a The moieties shown in blue, red and pink correspond to tris­(4-bromophenylamine), *p*-phenylenediamine and 2,5-dibromobenzoic acid, respectively.

**PTPA163** was initially synthesized using a Buchwald–Hartwig (B–H) cross-coupling reaction according to literature methods using toluene as solvent (S.3.3).^34,54^ The monomers, tris­(4-bromophenylamine), *p*-phenylenediamine and 2,5-dibromobenzoic acid (Scheme 3), were reacted in a 1:6:3 ratio, giving the 163 suffix in the name of this POP. This material was recently reported as an attractive candidate for efficient CO_2_ conversion and utilization compared with its nonfunctionalized analogue, improving CO_2_ uptake (273 K, 1 bar) from 2.16 to 3.44 wt %.^54^ We propose that the carboxylic acid functional group contributes to the hydrogen-bonding and dipole interactions,^34,35^ both with the solvent used for the synthesis of **PTPA163** and with the CO_2_ molecules during the gas adsorption application. However, to date limited discussion has been dedicated to the role of each interaction.

Bottom-up HSP prediction, *i.e.*, based on monomer structures, is impractical in this scenario because these monomers can combine in different ways, giving branched or cyclic substructures in the target material; the molecular structure is thus essentially unknown. *MLoc* treats the polymer matrix as an ensemble, allowing the HSPs to be obtained from a limited number of experimental measurements and processing through the *MLoc* algorithm.

The solubility score of **PTPA163** was generated from UV/vis absorbance measurements in a diverse set of solvents (hexane, ethyl acetate, acetonitrile, xylene, toluene, ethanol, 1,4-dioxane, diethyl ether, 1,2-dichloroethane, chloroform, acetone, isopropanol, tetrahydrofuran and water), using their UV/vis maximum absorbance values in the benzenoid band. We here note that, for materials such as **PTPA163** that exist multiple characteristic absorption bands (benzenoid and quinoid), UV/vis maximum absorbance values across test solvents should be taken consistently from the same absorption band. The partial HSPs of these test solvents covered a broad range in each dimension, to ensure unbiased sampling before calculating the HSPs of **PTPA163**. The UV/vis input for *MLoc*, including the experimentally measured UV/vis maximum absorbance values (Abs.) at the corresponding wavelength (λ_max_, in nm) and the HSPs of test solvents, alongside results predicted by *MLoc*, are summarized in Table 1.

**Table 1:** *MLoc*-Predicted HSPs of PTPA163^[*M*]^ and Comparison to the Hansen distance, *R*, of Test Solvents^a^

compound	Abs.^b^	λ_max_/nm^b^	δ_D_/ MPa^1/2^	δ_P_/ MPa^1/2^	δ_H_/ MPa^1/2^	*R*/ MPa^1/2^
**PTPA163** ^[*M*]^	N/A	N/A	16.81	5.78	7.96	0
tetrahydrofuran (THF)*	0.73	356	16.80	5.70	8.00	0.09
ethyl acetate	0.20	315	15.80	5.30	7.20	2.21
chloroform	0.53	371	17.80	3.10	5.70	4.03
acetone	0.21	344	15.50	10.40	7.00	5.39
1,4-dioxane*	0.07	320	19.00	1.80	7.40	5.95
diethyl ether	0.49	275	19.00	7.40	4.10	6.06
1,2-dichloroethane	0.14	370	14.50	2.90	5.10	6.15
xylene	0.03	300	17.60	1.00	3.10	7.00
toluene*	0.10	324	18.00	1.40	2.00	7.78
isopropanol	0.03	316	15.80	6.10	16.40	8.68
hexane	0.03	278	14.90	0.00	0.00	10.56
ethanol	0.48	369	15.80	8.80	19.40	12.00
acetonitrile	0.29	366	15.30	18.00	6.10	12.72
water	0.19	366	15.50	16.00	42.30	35.92

a Solvents labelled with * (THF, 1,4-dioxane and toluene) were used in further validation experiments.

b Abs. and λ_max_ are experimentally measured UV/vis maximum absorbance values (Abs.) at the corresponding wavelength (λ_max_) in the benzenoid band of each test solvent.

The HSPs of **PTPA163** were predicted as 16.81 MPa^1/2^ (δ_D_), 5.78 MPa^1/2^ (δ_P_), 7.96 MPa^1/2^ (δ_H_), respectively, with a global error of 0.01 MPa^1/2^ (obtained from rounding the convergence threshold used for the optimization, 0.005 MPa^1/2^ to the same number of decimal places as the HSPs). All solvent candidates were sorted according to the Hansen distance from **PTPA163** (by column *R*).

We highlight three solvents, tetrahydrofuran (THF), 1,4-dioxane and toluene (labeled with * in Table 1) that are commonly used for the synthesis of POPs via a B–H coupling reaction.^34,55^ The Hansen distances of THF (0.09 MPa^1/2^), 1,4-dioxane (5.95 MPa^1/2^) and toluene (7.78 MPa^1/2^) deviated at meaningfully different levels from the target polymers. No chemical compatibility issues were expected between these three solvents and other reagents in the synthetic route used, where sodium *tert*-butoxide (a strong base and an oxidizing agent) could have been a concern. **PTPA163** was synthesized in each of these three solvents, allowing us to compare the CO_2_ uptake performance of the resulting materials (Table 2).

**Table 2:** Solvent-Dependent CO_2_ Uptake in Weight Percentage (wt %) of PTPA163 Synthesized in Solvents of Interest^a^

solvent	*R*	Δδ_D_/ MPa^1/2^	Δδ_P_/ MPa^1/2^	Δδ_H_/ MPa^1/2^	CO_2_ uptake/wt %
THF	0.09	0.01	0.08	0.04	6.95 ± 0.35
1,4-dioxane	5.95	2.19	3.98	0.56	4.86 ± 0.24
toluene	7.78	1.19	4.38	5.96	3.44 ± 0.17

a Standard deviation of CO_2_ uptake is calculated over three repeated experiments.

According to the Hansen distance, THF is predicted as the best solvent, followed by 1,4-dioxane and toluene. **PTPA163** synthesized in THF resulted in the highest average CO_2_ adsorption (6.95 wt %), followed by 1,4-dioxane (4.86 wt %) and toluene (3.44 wt %), at 273 K, 1 bar (Table 2). The results agree with our hypothesis that better matching in terms of Hansen distance leads to better control of porosity and related properties (particularly the BET surface area as detailed in S3.5), hence higher CO_2_ adsorption capacity. We note that the best solvent identified by *MLoc* here (THF) achieves an approximate doubling in CO_2_ uptake compared to PTPA derivatives that have low CO_2_ uptake (e.g., **PTPA** (2.16 wt %)^54^ and **PTPA3** (2.20 wt %)).^56^ Compared with the initial synthesis of **PTPA163** using toluene,^56^ the CO_2_ uptake doubles, from 3.44 to 6.95 wt %, achieved by systematically lowering the Hansen distance (*R*) of solvents.

On confirming the significance of the Hansen distance (*R*), we then explored the partial HSPs. We noted that all three HSPs of THF are closely matched to **PTPA163**. The hydrogen-bonding difference (Δδ_H_) is similar for THF (0.04 MPa^1/2^) and 1,4-dioxane (0.56 MPa^1/2^), both significantly smaller than the value for toluene (5.96 MPa^1/2^), whereas the dipolar difference (Δδ_P_) of THF (0.08 MPa^1/2^) is comparably smaller than both 1,4-dioxane (3.98 MPa^1/2^) and toluene (4.38 MPa^1/2^). This supports the earlier proposal that both dipolar and hydrogen-bonding interactions contribute to the observed solvent effects on CO_2_ uptake; if only Δδ_H_ was important, we would expect a better result for 1,4-dioxane.

Applying the *MLoc* workflow, we then constructed the first HSP database for POPs: the HSP–POP database. As noted earlier, a full synthesis–characterization cycle for POPs typically takes between 1 day and 1 week,^39^ causing a bottleneck for obtaining fully characterized materials to construct their property databases. This development aims to address the lack of published data for POPs and to further support the exploration of the link between solvents and material properties in this field. The development and initial exploration of the HSP–POP database are detailed in the next section.

#### Data Generation for Porous Organic Polymers Using *MLoc*: the HSP–POP Database

The HSP–POP database (available as ESI and on GitHub as “HSP_POP_full_database.csv”) collects data for 17 POPs studied experimentally between 2019 and 2022, derived from 14 monomers shown in Scheme 4 and Table S.8. Conventional bottom-up HSPs prediction is unavailable in these cases, as the detailed molecular structure cannot be determined, as noted for **PTPA163** (*vide supra*). The building blocks, stoichiometric ratios and the HSPs predicted by *MLoc* are listed in Table 3. All reactions follow the same mechanism as **PTPA163** (B–H coupling) except Group 16, where a polycondensation reaction was used.^57,58^ This database also includes data from over 80 screening reactions, varying solvents for synthesis and purification, as well as additives, catalysts, catalyst loadings and monomer ratios. Given the sensitivity of polymerization with respect to reaction conditions, these may all have an impact on the reaction outcomes, including yield, morphology and functionalities, of the POPs prepared. The details of representative synthesis procedures for each POP and input data used for *MLoc* are provided in S.3.6. The full data in this HSP–POP database are included in S.4 (ESI).

**Scheme 4:**
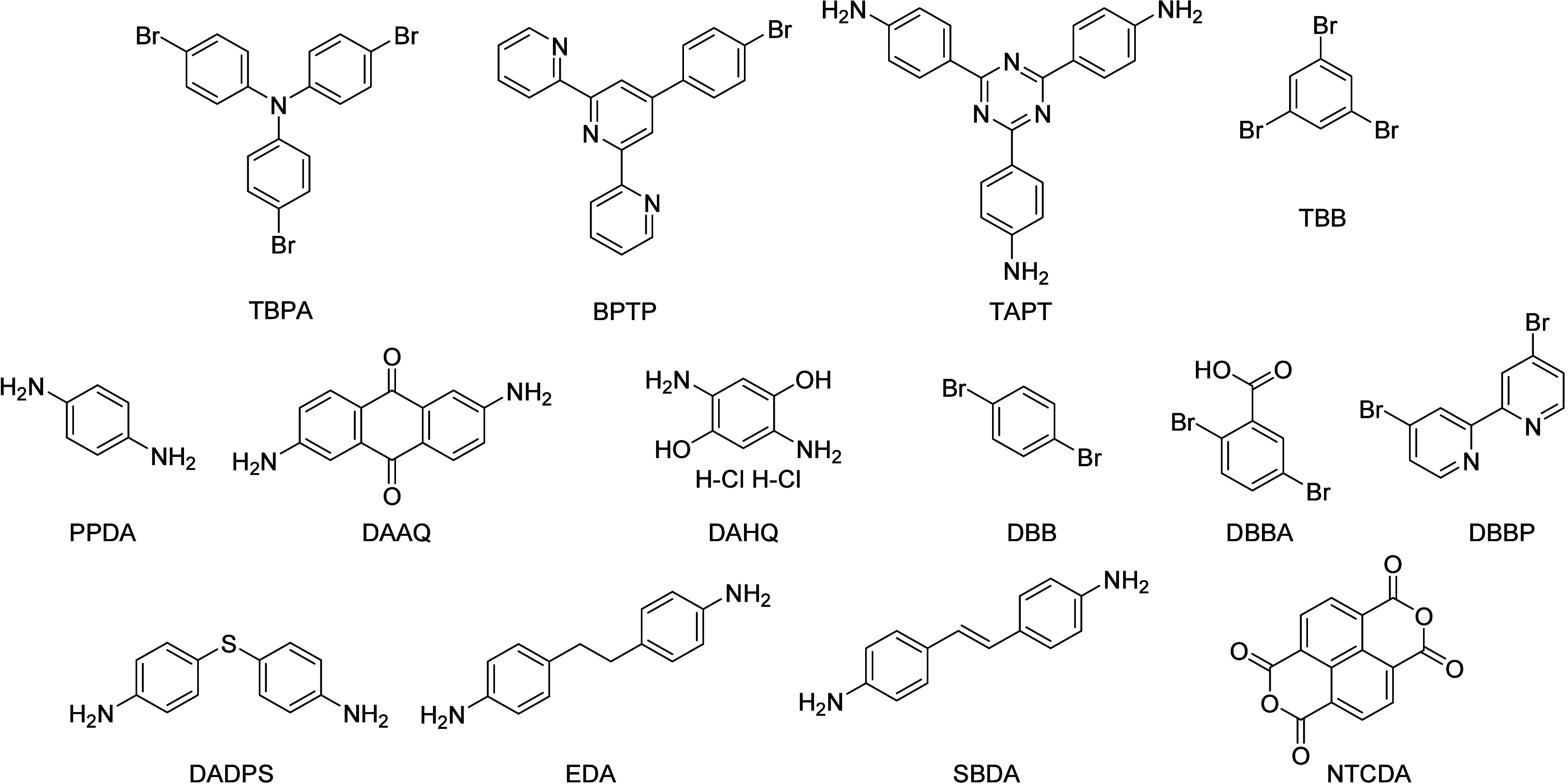
Chemical Structures of Building Block in the HSP-POP Databases^a^

a Building blocks of the current database include tris­(4-bromophenyl) amine (**TBPA**), 4,4’-ethylenedianiline (**EDA**), 4,4’-stilbenediamine (**SBDA**), p-phenylenediamine (**PPDA**), 4,4’-diaminodiphenyl sulfide (**DADPS**), 1,4-dibromobenzene (**DBB**), 2,5-dibromobenzoic acid (**DBBA**), 4’-(4-bromophenyl)-2,2’:6’,2’’-terpyridine (**BPTP**), 4,4’-dibromo-2,2’-bipyridine (**DBBP**), 2,6-diaminoanthraquinone (**DAAQ**), 1,3,5-tribromobenzene (**TBB**), 2,5-diaminohydroquinone dihydrochloride (**DAHQ**), tris­(4-aminophenyl)­triazine (**TAPT**), 1,4,5,8-naphthalenetetracarboxylic dianhydride (**NTCDA**).

**Table 3:** *MLoc*-Calculated HSPs for 17 POPs^a^

group	name	block 1	block 2	block 3	ratio	δ_D_/MPa^1/2^	δ_P_/ MPa^1/2^	δ_H_/ MPa^1/2^	synthesis details (ESI)
1	PTPA-1O	TBPA	EDA	-	1/1.5	17.85	4.84	5.80	S.3.6.1
2	PTPA-2O	TBPA	SBDA	-	1/1.5	16.97	5.97	7.78	S.3.6.2
3	PTPA-3O	TBPA	PPDA	-	1/1.5	16.66	7.78	9.51	S.3.6.3
4	PTPA-4O	TBPA	DADPS	-	1/1.5	17.00	5.89	7.43	S.3.6.4
5	PTPA-4O–U1A1	TBPA	DADPS	DBB	1/6/3	16.94	6.58	8.20	S.3.6.5
6	PTPA-4O–U1B1	TBPA	DADPS	DBB	2/5/2	17.71	6.61	7.03	S.3.6.5
7	PTPA163	TBPA	PPDA	DBBA	1/6/3	16.81	5.78	7.96	S.3.3 (case study)
8	CMP-TP-1	TBPA	BPTP	PPDA	9/1/15	17.85	13.11	8.85	S.3.6.6
9	CMP-TP-4	TBPA	BPTP	PPDA	6/4/15	17.64	12.99	9.17	S.3.6.7
10	CMP-BP-1	TBPA	DBBP	PPDA	9/1/15	17.82	11.84	8.70	S.3.6.8
11	CMP-BP-4	TBPA	DBBP	PPDA	6/4/15	17.79	12.13	7.70	S.3.6.9
12	PPAAQ	TBPA	DAAQ	-	1/1.5	17.75	11.64	8.27	S.3.6.10
13	PBAQ	TBB	DAAQ	-	1/1.5	17.86	12.11	9.24	S.3.6.11
14	PPAHQ	TBPA	DAHQ (2HCl)	-	1/1.5	17.65	11.91	9.37	S.3.6.12
15	PBHQ	TBB	DAHQ (2HCl)	-	1/1.5	17.65	12.48	9.78	S.3.6.13
16	NPI-4	TAPT	NTCDA	-	1/1.5	18.17	12.14	9.19	S.3.6.14
17	PTPA-3	TBPA	SBDA	-	1/1.5	17.50	12.70	10.50	S.3.6.15

a See Scheme 4 for structures and abbreviations.

The data gathered in this HSP-POP database have provided further insights and new knowledge of the role of solvents and other reaction variables in the preparation of POPs, serving as a guide to controlling the synthetic conditions in such multivariable systems. For instance, by evaluating the reaction conditions and outcomes of six POPs (group 7, 8, 9, 10, 11, 17 in Table 3), synthesized in the following solvents: DMSO, propylene carbonate, ethanol, toluene, THF, 1,4-dixoane and DMF, we noted that, in addition to CO_2_ uptake, the yield of POPs synthesized by B–H coupling reactions, even with different building blocks, is sensitive to the Hansen distance (Table S.25).

Intriguingly, an optimal Hansen distance of ca. 8.5 MPa^1/2^ was found to correlate with the best yield in each group (Figure 3a). This optimal Hansen distance likely represents the radius of the Hansen sphere (*R*_0_) for general POPs (Figure 3b), which has not been published before for POPs, yet matches well with an empirically accepted threshold for good solvents and nonsolvents.^59^ Solvents within the Hansen sphere for a reaction are typically found to be good solvents, whereas those located outside the sphere are considered poor solvents.^48^ While earlier research primarily concluded that a larger Hansen distance can hinder polymerization (C in Figure 3b),^34^ Figure 3a suggests that there is a trade-off between lower isolated yield and a smaller Hansen distance (A in Figure 3b) which also needs to be considered in materials synthesis, even though a lower Hansen distance contributes to better control over CO_2_ uptake as noted earlier in the case study. The optimal Hansen distance lies between these two scenarios (B in Figure 3b) and this trade-off should be considered when scaling up the synthesis of this type of POPs.

**Figure 3:**
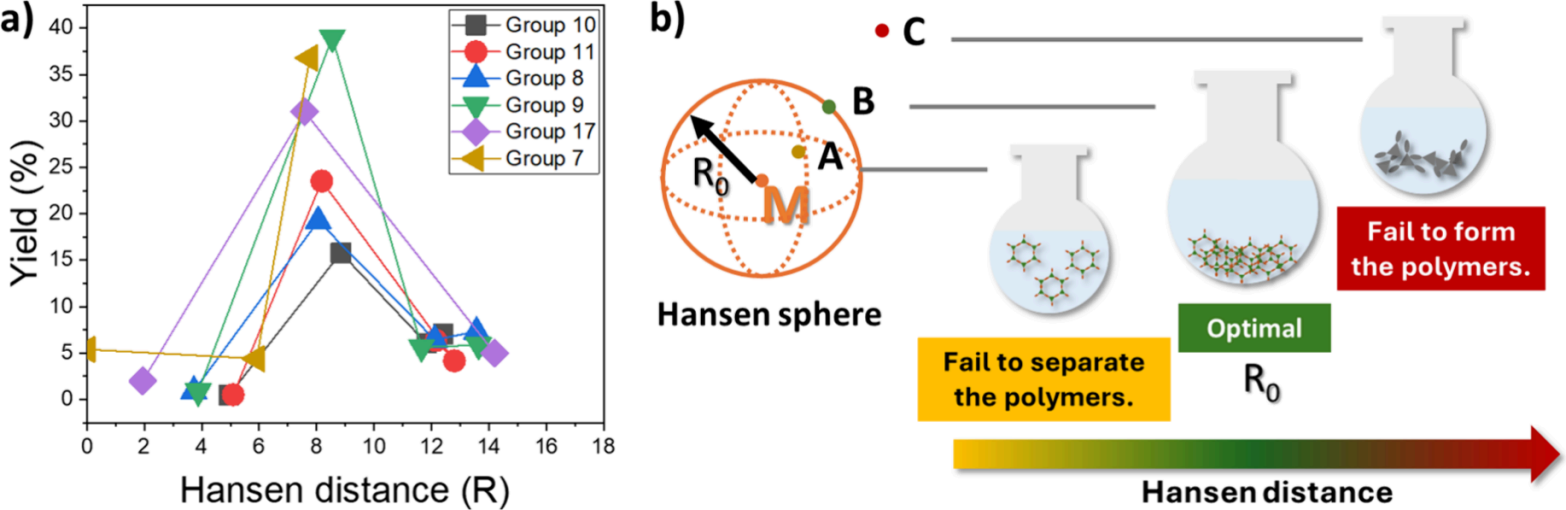
(a) Comparison of the yield of group(s) 7, 8, 9, 10, 11, and 17 with respect to the Hansen distance of tested solvents and (b) proposed explanations using the Hansen sphere.

While this database of HSPs offers a useful framework for consideration of solvent effects on the synthesis and properties of POPs, we also acknowledge that the impact of solvent choice on reaction outcomes and the properties of materials can be complicated and case dependent.^60^ To support future development, we have included additional solvent features and SMILES notation in the *MLoc* database. We anticipate that these extended features, where applicable and when data permit, can support data-driven research aimed at uncovering deeper solvent effects.

We also note that benchmarking of this approach against HSPs obtained by alternative methods is challenging, both because not many reliable measurements of polymer solubility have been reported, and because there were problems with previous approaches, which have been addressed in the present work. We have been able to compare data for graphene, which has been included in SI Section S.5 to demonstrate the validity of *MLoc*. There, we have also discussed expected differences from reported HSPs.

## Conclusions

In this work, we have presented a solvent selection framework, *MLoc*, for efficient predictions of HSPs for novel materials based on UV/vis spectroscopic data, which can be determined routinely. This algorithm does not need extensive data sets, explicit structural information, or established knowledge bases, effectively addressing major challenges faced by current quantitative solvent selection methodologies. In addition, the deconvolution of Hansen distances into HSPs allows the interpretation of data, guiding the design of reaction conditions.

By implementing *MLoc*, we have determined the HSPs of a novel porous organic material and demonstrated how solvent selection can be used to enhance material properties. The *MLoc* framework, for the first time, enabled the use of HSP theory to establish a solvent–morphology–CO_2_ uptake relationship for POPs.

We have also used *MLoc* to construct, to the best of our knowledge, the first HSP–POP database from comparatively limited experimental data. This database provided initial insights into solvent design principles by identifying an optimal Hansen distance for POP synthesis.

While this goes beyond the scope of this work, we propose that the *MLoc* algorithm can be extended to a broader range of materials, including but not limited to other POPs. For example, we have recently applied the *MLoc* routine to support selective phase separation research on graphene.^61^

We anticipate that the *MLoc* workflow will benefit materials discovery at the intersection of experimental chemistry, chemoinformatics and digital chemistry, and we encourage further contributions to the HSP–POP database alongside its broader applications beyond POPs.

## Software and Code Development

*MLoc* is freely available under a GPL 3.0 license at: https://github.com/xueannafang/hsp_mloc_v2

*MLoc’s* built-in database is curated from *PubChem* and the HSPs handbook,^48^ with common synonyms, physical properties and SMILES strings included (Scheme S.3 in S.2.1). The current version summarizes information for 249 solvents. Further details of the database features and how to run the algorithm, as well as sample outputs can be found in Section S.2 of the ESI.

## Experimental Methods

### Chemicals

Chemicals and reagents used in the case study were 1,4-dioxane, xylenes, hexane, ethyl acetate, diethyl ether, ethanol, toluene, dichloroethane, chloroform, acetone, isopropanol, tetrahydrofuran, acetonitrile, deionized water, sodium fluoride and sodium chloride. Full supplier information is listed in Table S.5 in S.3.1.

### Synthesis and Characterization

**PTPA163** used in the case study was synthesized, purified and characterized following a literature method,^54^ described in detail in S.3. All syntheses were performed at the boiling point of each solvent, with yields provided in Table S.6. Synthesis procedures of POPs in the HSP–POP database are provided in S.3.6.

## Supplementary Material





## Data Availability

The data underlying this study are available in the electronic Supporting Information documents. The Supporting Information for this manuscript contains additional information on the underlying theory, further details of *MLoc* and the experimental work reported, as well as outlining the structure of the HSP-POP database and relevant references. The full database described in this work has been included as an Excel database. All code and databases underlying this study are openly available on GitHub and via Zenodo at the following URLs: https://github.com/xueannafang/hsp_mloc_v2; DOI: 10.5281/zenodo.15383018. https://github.com/xueannafang/hsp_toolkit_prototype; DOI: 10.5281/zenodo.15383058. https://github.com/xueannafang/HSP_toolkit_docs; DOI: 10.5281/zenodo.15383045.

## Footnotes

a We note that the optimization function of eq 4 can be proven to be convex, so there exists a single, global minimum (see SI Section S1.9).

## Notes

Supplementary theory details, further details of *MLoc,* additional experimental details, outline of the structure of the HSP-POP database, benchmarking of prediction, and relevant references (PDF)
Full database described in this work included as an Excel database (XLSX)
