# Supplementary material for: A Solvent Selection Framework for Porous Organic Polymers
Source: J Chem Inf Model. 2025 Nov 4;65(22):12377–86. doi: 10.1021/acs.jcim.5c02163 (PMC12648659; doi:10.1021/acs.jcim.5c02163)
Supplement: Supplementary file 1 [file ci5c02163_si_001.pdf]

Supporting Information

A solvent selection framework for porous organic polymers

Xue Fang[a,c], Ulzhalgas Karatayeva[a], John D. Worth[a,b], Merve Gumussoy Girgin[a], Safa Ali Al Siyabi[a], Dauren Mukhanov[a], Ella M. Gale[a], Charl F. J. Faul[a,*], and Natalie Fey[a,*]

[a] School of Chemistry, University of Bristol, Cantock's Close, Bristol, BS8 1TS, UK. *Corresponding authors, Email: Charl.Faul@Bristol.ac.uk; Natalie.Fey@Bristol.ac.uk

[b] Bristol Composites Institute, School of Civil, Aerospace and Mechanical Engineering, University of Bristol, University Walk, Bristol, BS8 1TR, UK

[c] Department of Chemistry, Molecular Sciences Research Hub, Imperial College London, 82 Wood Lane, London, W12 0BZ, UK

Further information:

The full database described in this work has been included as an Excel database (MLoc_HSP_POP_full_database.xlsx).

All code and databases underlying this study are openly available on GitHub and via Zenodo at the following URLs:

https://github.com/xueannafang/hsp_mloc_v2; https://doi.org/10.5281/zenodo.15383018

https://github.com/xueannafang/hsp_toolkit_prototype; https://doi.org/10.5281/zenodo.15383058

https://github.com/xueannafang/HSP_toolkit_docs; https://doi.org/10.5281/zenodo.15383045.

Contents

Supporting Information 1
1. Supplementary section for theories 3
1.1. Evaluation of existing solvent selection methods 3
1.2. The linear solvation energy relation (LSER) 4
1.3. Hildebrand and Hansen solubility parameters: deconvolution of the solvent–polymer interactions 5
1.4. Estimating solubility parameters of polymers by UV/Vis absorbance 6
1.5. The statistical method of HSPs estimation and its application in practice 7
1.6. Explanation of the failure of one-to-one mapping between δT and HSPs 8
1.7. Comparison of *MLoc* with random guess initialization 9
1.8. Explanation of the conceptual similarity and differences of *MLoc* with k-means 10

1.9. Proof of the convexity of the optimization function..11
2. Supporting information for *MLoc*...12
2.1. Built-in database...13
2.2. Preparation of polymer suspensions and UV/Vis data.....................................14
2.3. Preparing an input document for solvent candidates.....................................15
2.4. Loading input documents...16
2.5. Setting hyperparameters for gradient descent..17
2.6. Setting output filename..17
2.7. Running *MLoc*..17
2.8. Anticipated outcomes...18
2.9. Troubleshooting..20
3. Supporting information for experimental work..21
3.1. Chemicals...21
3.2. Instrumental details and methodologies for characterization.......................22
3.3. PTPA163 synthesis and characterization..23
3.4. Discussion of the contribution of partial HSPs for PTPA163..........................29
3.5. Full data for PTPA163 predicted by *MLoc*..29
3.6. Experimental details and UV/Vis data for POPs in the HSP-POP database....................30
3.7. Solvent effect on yield for Group 7 to 11 and Group 17 – Screening results.................41
4. Database structure of the HSP-POP database..41
4.1. The key information included in the HSP-POP database...............................41
4.2. The “Overview” tab...42
4.3. The “Building_blocks” tab..43
4.4. The “MLoc_ip_data” tab...43
4.5. The “MLoc_best_solv” tab..43
4.6. The “MLoc_full_detail” tab...43
4.7. The “reaction_details” tab..45
5. Benchmarking and comparison with reported methods.......................................46
References..47

1. Supplementary section for theories

1.1. Evaluation of existing solvent selection methods

Multivariate empirical solvation models such as the Linear Solvation Energy Relationships (S.1.2)[1] are comparatively easy to interpret, but they rely heavily on solute-specific experimental data. This reliance becomes a major limitation for novel systems, where significant experimental work is required for data curation. In contrast, bottom-up predictions such as via group contributions[2] or deep-learning methods[3] can simplify experimental efforts, but these methods typically rely on explicit 2D or 3D molecular representations of information on atoms and their connections. While such methods work well for molecules with well-defined components, they can fail for complex systems, such as oligomer–polymer mixtures or composite materials, where molecular representations are not easily available. Dimensionality reduction methods, such as the solvent map generated by principal component analysis (PCA) of a database of solvent descriptors reported by Murray and co-workers,[4–6] offer an efficient way to reduce the number of solvent descriptors. However, these methods focus more on *designing* screening experiments rather than directly *predicting* the optimal solvents for a specific problem, and thus still require experimental screening. As models become more complex, challenges related to the quality of training data and model interpretability can emerge. The sophistication of ML models, in terms of hidden variables and hyperparameters, can obscure the underlying mechanisms and make it difficult to extract mechanistic insights for the target problem. This trade-off between accuracy, simplicity and interpretability remains an obstacle in the development of a universal solvent selection method.

We evaluate these methods and their deviation from an ideal model (*i.e.*, the goal of this work) in Scheme S. 1, where we provide a checklist that addresses benefits for both users and developers. This checklist assesses the reliance on solvent and solute databases, reliance on explicit structural information, experimental efforts, computational costs, and interpretability. Methods are rated as positive, neutral, or negative in each criterion, represented by green, amber, and red, respectively.

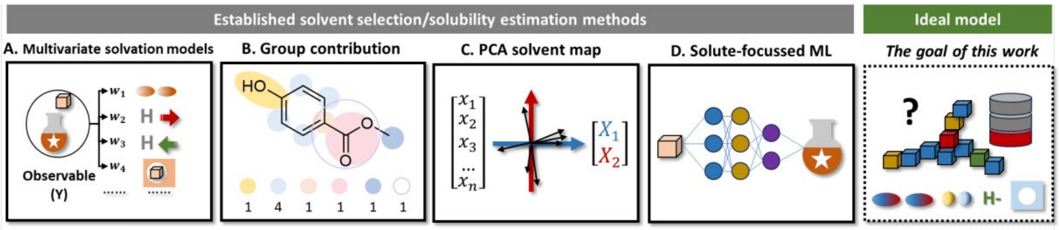

Criteria (for users)	A	B	C	D	Goal
Reliance on solvent databases	High	Low	Moderate	Low	Low
Reliance on solute databases	High	Low	Moderate	Low	Low
Reliance on structural information	Low	High	Low	High	Low
Computing cost	Low	Low	Low	High	Low
Experimental efforts	High	Low	Moderate	Low	Low
Interpretability	High	Moderate[a]	Low	Low	High

Criteria (for developers)	A	B	C	D	Goal
Reliance on solvent databases	N/A	High	High	High	Moderate
Reliance on solute databases	N/A	High	Low	High	Low
Reliance on structural information	N/A	High	Low	High	Low
Training cost	N/A	Low	Low	High	Low

Scheme S. 1 Comparison of established solvent selection/solubility estimation strategies for users and developers. (The positive, neutral and negative performance is labelled in green, amber and red, respectively.) [a] The interpretability of group contribution may depend on the explicitness of molecular representations and how strictly the properties to be modelled follow the summation of individual molecular fragments.

Multivariate empirical solvation models (A in Scheme S. 1), such as the linear solvation energy relationships (S.1.2),[1] are built on well-defined physiochemical solubility parameters (*e.g.*, polarizability, hydrogen-bonding donor or acceptor ability) derived from experimental measurements, making them comparatively easy to interpret. However, these models depend heavily on solute-specific experimental data, requiring significant experimental work for novel systems. The group contribution method (B in Scheme S. 1) addresses this transferability problem by predicting molecular properties through contributions of individual functional group, typically represented by 2D or 3D descriptors. This approach retains a level of interpretability as the contribution of functional groups is tested on experimental databases, but can still be affected by the molecular representations, as well as how strictly the properties to be modelled follow the linear summation of the properties of individual fragments. The other bottleneck is to precisely deconstruct target molecules into appropriate fragments, as well as the need to introduce higher-order corrections based on heteroatom content and level of conjugation.[2] Consequently, this method is heavily parameterized and often restricted to small molecules.[7] Moreover, the dependence on explicit molecular structure makes it unsuitable for materials such as polymers and composite materials as noted earlier, where molecular representations are either too complex or unavailable.

To simplify the parameterization of solvents, dimensionality reduction techniques such as PCA have been utilized (C in Scheme S. 1).[4–6] A promising outcome is the three-dimensional (3D) PCA solvent map reported by Murray and co-workers that allows visualization of over twenty solvent features in only three variables.[8] Interpretation becomes more challenging in this case due to the linear transformation used to project the high-dimensional feature space into fewer dimensions. In addition, this map was mainly intended for designing experimental solvent screens instead of direct prediction of the best solvents for a specific problem. The contribution of solutes is not considered, meaning experimental solvent screening is still required.

In contrast to reducing the number of descriptors for visualization, a different approach is to leverage big data and surrogate models to make solvent behavior predictable for any given solute. We broadly refer to this approach as solute-focused ML (D in Scheme S. 1).[3,9–12] High accuracy (93%) binary solubility classification (soluble/insoluble) has been reported for polymers using deep-learning methods.[3] Predictions for continuous solubility parameters of small molecules and oligomers (fewer than five monomers) have also been achieved using Bayesian approaches.[13] Similar to group contributions, explicit structural information is necessary, and more computationally-demanding information, such as surface charge density or 3D molecular descriptors, can be required to enhance accuracy.[13] While this approach offers improvements in user convenience, it also incurs high computational and training costs, as it relies on large, high-quality datasets that comprehensively sample the relevant chemical space. The interpretability is another limitation. As models become more complex, additional hidden variables and hyperparameters emerge, making it harder to understand how the model arrives at its conclusions. This opacity can be a drawback when seeking mechanistic insights for the problem considered.

1.2. The linear solvation energy relation (LSER)

The linear solvation energy relation (LSER) was reported by Taft *et al.*, where the solvent effect is deconvoluted into a cavity term and three solvatochromic terms (Eq. S. 1).[1]

$$P = P_0 + c_1 \frac{{\delta_T^{(s)}}^2}{V_M^{(m)}} + c_2 \frac{\pi^{*(s)}}{\pi^{*(m)}} + c_3 \frac{HBD^{(s)}}{HBA^{(m)}} + c_4 \frac{HBA^{(s)}}{HBD^{(m)}} + e \qquad \textit{Eq. S. 1}$$

In LSER (Eq. S. 1), P and P_0 represent solvent-dependent properties, such as reaction rates, absorption energies, partition coefficients, etc.,[1] with P_0 being the standard reference measured in cyclohexane. δ_T is the Hildebrand solubility parameter,[14] which, together with the molar volume (V_M), construct the solute cavity term. π^*, HBD and HBA, are the Kamlet-Taft solvatochromic parameters,[15–20] and represent polarizability, hydrogen bond donor ability and hydrogen bond acceptor ability, respectively. The superscript (s) and (m) stands for the corresponding parameter of solvent and solute, respectively. Each term is associated with a linear coefficient (c_i), with an error term (e) containing residues for systems that require extra corrections.

1.3. Hildebrand and Hansen solubility parameters: deconvolution of the solvent–polymer interactions

The theories that are foundational to the present work are the Hildebrand and Hansen solubility theories and associated solubility parameters.[21] Both theories are widely applied in modern polymer science due to their consideration of the volume of solute molecules. The Hildebrand solubility parameter (denoted as δ_T in this work) represents a milestone arising from the proposal of the general law of vapour pressures of ideal solvents (Raoult's law),[22] where non-ideal solvent behavior began to be addressed for the first time.[21] The Hildebrand solubility parameter describes the solvent–solvent interactions interrupted by solute cavities. Large molecules, particularly polymers, can induce cavities in a continuum solvent phase, which can affect the solvation energy by contributing to the entropy change.[23] The Hildebrand solubility parameter is defined as the volume density of cohesive energy (Eq. S. 2)[23]:

$$\delta_T = \sqrt{\frac{\Delta H - RT}{V}} \qquad \textit{Eq. S. 2}$$

where ΔH is the molar heat of evaporation ($\mathrm{kJ/mol}$); V is the molar volume ($\mathrm{cm^3/mol}$), R is the ideal gas constant and T is the absolute temperature (K).

The absolute difference between δ_T of two substances represents their affinity, $\Delta\delta_T$ (Eq. S. 3), which has been utilized in the BXJ approach (see manuscript for further details) to describe the quality of solvents.[24] A lower $\Delta\delta_T$ between polymer/solute (1) and solvent (2) indicates better compatibility.

$$\Delta\delta_T = |\delta_T^{(1)} - \delta_T^{(2)}| \qquad \textit{Eq. S. 3}$$

The Hildebrand solubility theory initially focused on London dispersion forces,[25] which was later refined by Burrell, Blanks, Prausnitz and Hansen *et al.* to include permanent dipole–dipole forces and hydrogen-bonding forces.[25–27] In the 1960s, the concept of 3D parameters named after Hansen was proposed (Eq. S. 4),[28]

$$\delta_T^2 = \delta_D^2 + \delta_P^2 + \delta_H^2 \qquad \textit{Eq. S. 4}$$

where the subscripts D, P and H, stand for dispersion, dipolar and hydrogen-bonding components, respectively. Individually δ_D, δ_P, δ_H have been referred to as "*partial HSPs*". The unit of each parameter is $\mathrm{MPa^{1/2}}$. (To avoid confusion, we use "*HSPs*" to refer the Hansen solubility parameters, only, for short; the "*Hildebrand solubility parameter*", which occasionally is also referred to as "*total Hansen solubility parameter*", will be referred to by its full name or δ_T in the rest of this paper.)

Eq. S. 3 and Eq. S. 4 are theoretical strategies to quantify the concept of "like dissolves like" and to deconvolute a one-dimensional scale into three orthogonal contributions. This theory and approach are still being developed and efforts continue to validate them in diverse experimental settings.[29] Here we assume that this approach provides an efficient framework to approximate complicated solvent–solute interactions when training data are limited.

Each partial HSP can be separately considered to yield the differences (in terms of $\Delta\delta_D$, $\Delta\delta_P$ and $\Delta\delta_H$) between two substances. The advantage is that this approach allows more precise control over solvent behavior by targeted manipulation of each partial interaction.

The dispersion interaction is important in large, non-polar systems,[30–32] and has been applied in surface and supramolecular science, for example, to control physisorption and molecular recognition.[33,34] Dipolar and hydrogen-bonding interactions are both critical in polar systems. Endowing molecules with hydrogen-bonding moieties is a facile way to induce directional and reversible interactions,[35] which are essential for tuning the functionalities of materials.[36–39]

In Hansen's theory, the overall compatibility between two substances is quantified by the Hansen distance, R, as defined by Eq. S. 5. The concept is analogous to $\Delta\delta_T$ in that a lower R value indicates better compatibility.

$$R = \sqrt{4\left(\delta_D^{(1)} - \delta_D^{(2)}\right)^2 + \left(\delta_P^{(1)} - \delta_P^{(2)}\right)^2 + \left(\delta_H^{(1)} - \delta_H^{(2)}\right)^2} \qquad \text{Eq. S. 5}$$

It needs to be noted that the Hansen distance is commonly compared with the radius of the *Hansen sphere*, which is a solute-specific threshold to distinguish between good and poor solvents.[40] The determination of the Hansen sphere typically relies on commercial software *HSPiP*, using binary solubility indicators.[41] This workflow can nevertheless cause ambiguity when categorizing solubility, a continuous variable, into *soluble* or *insoluble*, as a binary score, hence affecting the fidelity and accuracy of the prediction. The design principles of this work (the *MLoc* workflow) are therefore exclusively based on the Hansen distance, and independent from the generation of the Hansen sphere.

In the BXJ approach, good solvents are expected to delay phase separation during the reaction, facilitating the formation of extended polymer networks. In contrast, poor solvents induce early stage phase separation, resulting in short oligomers that fail to form polymer networks.[42–44] Dipole–dipole and hydrogen-bonding interactions between solvents and reaction intermediates can be altered by varying solvents, consequently affecting their Hildebrand and Hansen solubility parameters, hence impacting the polymerization progress.[24]

1.4. Estimating solubility parameters of polymers by UV/Vis absorbance

To represent solvation energy using experimental observables, the UV/Vis absorbance has been widely accepted as a solubility indicator according to the Beer–Lambert law (Eq. S. 6),[45]

$$A = \varepsilon c l \qquad \text{Eq. S. 6}$$

where A is the absorbance (arbitrary unit), ε is the molar extinction coefficient associated with solutes ($\mathrm{M^{-1}cm^{-1}}$), c is the concentration (M); l is the optical path length (cm). Better solvents result in higher concentrations of dissolved solutes (c), hence stronger absorbance (A).

The absorbance is a direct indicator for the concentration of soluble fractions in the system investigated. Quantitative solvent—property relationships, such as the Linear Solvation Energy Relationship (LSER),[1] have been developed based on UV/Vis absorbance measurements.

The key advantage of using UV/Vis absorbance data is that the explicit chemical formulae of the solute molecules are not required. The UV/Vis absorbance has therefore been utilized to estimate solubility parameters of polymers.[24,46] The method was referred to as a *statistical method*, as reported by Ata *et al.* in their study of conductive carbon nanotubes.[46] Mixtures of polymers can be treated as a statistically averaged ensemble, without requiring explicit chemical formulae.

This method was also reported in 2019 to optimize the structure and functionality of POPs as part of the BXJ approach,[24] which serves as another foundation of this work for the generation of solubility data for target POPs: In the BXJ approach, the maximum absorbance of polymer supernatants in 10 to 15 solvent candidates is fitted to the solubility parameters of the corresponding solvents using a B-spline curve. The maximum of the fitting curve corresponds to the solubility parameter of the target material (S.1.5).

This B-spline fitting-based statistical method, although it works well for the estimation of the Hildebrand solubility parameter (δ_T), is not strictly applicable to HSPs. δ_T and HSPs do not follow one-to-one mapping, *i.e.*, one single δ_T can arise from more than one combination of HSPs (S.1.6) due to the expansion of the solubility space from 1D to 3D.[40] The problem therefore evolves from a 1D to a 3D multi-objective optimization scenario that requires higher-order methods to locate the optimum, which the *MLoc* workflow in this project addresses.

1.5. The statistical method of HSPs estimation and its application in practice

The workflow of the B-spline statistical method (applied in the BXJ approach) is illustrated in Scheme S. 2. This method utilizes UV/Vis absorbance to represent the solubility of polymer suspensions,[24,46] which has been grounded by the Beer–Lambert law (S.1.4).[45]

After completing the synthesis with standard literature-suggested protocols (Step 1),[24] the UV/Vis absorbance (abs.) of polymer suspensions in 13 solvents is recorded as the solubility indicator (Step 2). δ_T of common solvents are acquirable from a standard database,[40] whereas data for POPs is limited. To estimate the δ_T of POPs, the absorbance versus δ_T of each solvent is fitted using a B-spline curve (Step 3). $\Delta\delta_T$ can then be calculated according to Eq. S. 3 (Step 4) to support the solvent optimization (Step 5).

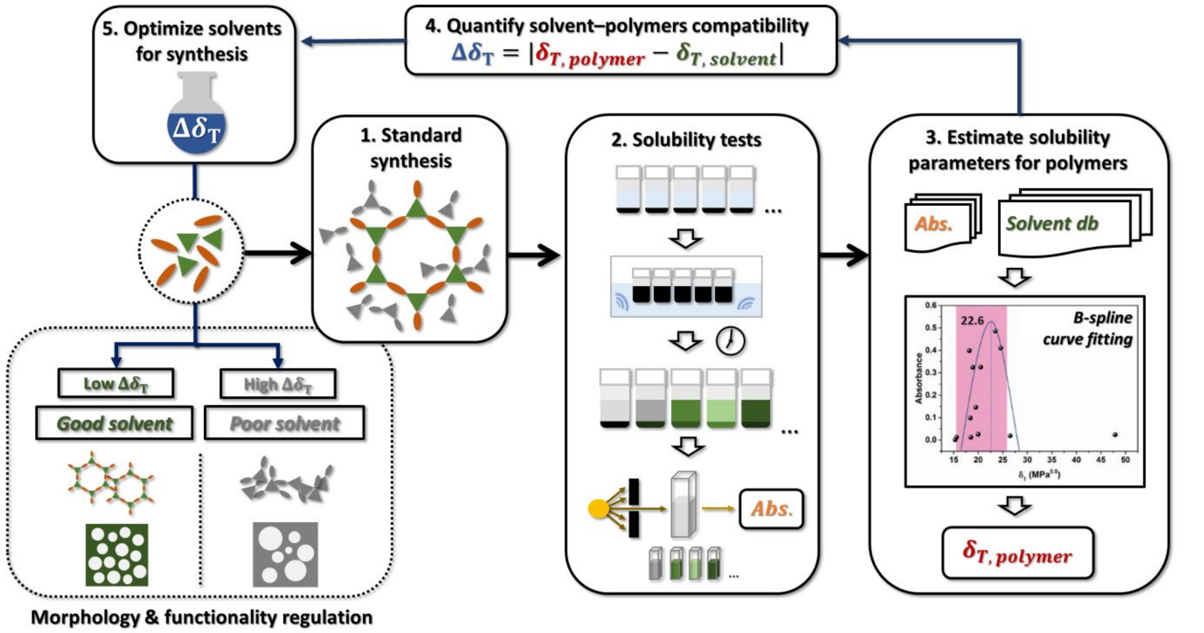

Scheme S. 2 Workflow of the BXJ approach.[24] Step 1: Synthesize the initial batch of polymers using standard literature methods. Step 2: Measure UV/Vis absorbance of saturated polymer supernatants. Step 3: Estimate solubility parameters of the polymer using B-spline fitting. Step 4: Calculate the difference between δ_T of the polymer and each test solvent. Step 5: Optimize the solvent condition by varying $\Delta\delta_T$.

Note that the BXJ approach has also been tried for the prediction of HSPs with the B-spline statistical method. However, the theory is not strictly appliable due to the lack of one-to-one mapping between δ_T and HSPs. This has been discussed in the main text and detailed below.

1.6. Explanation of the failure of one-to-one mapping between δ_T and HSPs

As addressed in the main text, HSPs are a 3D expansion of δ_T. The equivalent concept of δ_T is only comparable to the Hansen distance (R) rather than individual HSPs (δ_D, δ_P, δ_H).

An example given by Hansen are the dramatically different individual HSPs of ethanol and nitromethane, regardless of the similarity in δ_T. This arises because the scale and direction of individual HSPs do not always overlap, even when the sums of squared HSPs are the same.

The difference in the dimensionality of solubility space can lead to multiple combinations of HSPs predicted with the current B-spline statistical method, which requires extra effort to confirm the correct result.

The lack of one-to-one mapping can further cause errors in the prediction of the compatibility between two substances. An example is given in Figure S. 1, the coordinates of points A, B, C in the Hansen space are (2, 4, 3), (4, 2, 3), (3, 2, 4). The same δ_T is expected, 5.4 MPa$^{1/2}$. According to Eq. S. 3, $\Delta\delta_T$ is only calculated by the difference of each δ_T, the $\Delta\delta_T$ between AB, BC, AC hence are all 0 MPa$^{1/2}$. The conclusion is inconsistent with that predicted by the Hansen distance (Eq. S. 5), which yields R_{AB}, R_{BC}, R_{AC} equal to 4.47 MPa$^{1/2}$, 2.24 MPa$^{1/2}$ and 3.00 MPa$^{1/2}$, respectively.

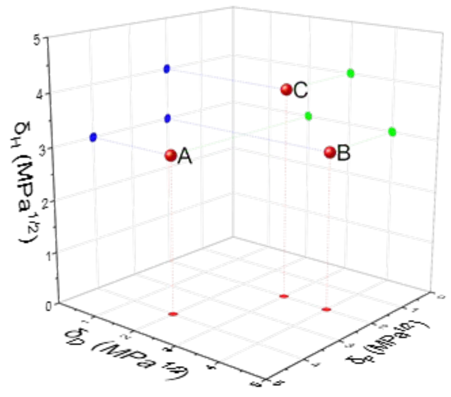

Figure S. 1 Illustration of discrepancy between three points with same δ_T whilst different individual HSPs.

1.7. Comparison of *MLoc* with random guess initialization

We considered the computing efficiency and adapted the initialization function of *MLoc* using numerical strategies, as detailed in the manuscript. We compared the outcome of *MLoc* with random guess initialization using a grid search method (Figure S. 2). The default convergence criterion (0.005 $MPa^{1/2}$) and learning rate (0.01) are applied, using the same input data provided in Table 1 in the main manuscript.

The results demonstrate that the *MLoc*-predicted values of δ_D (16.81 $MPa^{1/2}$) and δ_P (5.78 $MPa^{1/2}$) correspond to the global minima in their respective parameter space (Figure S. 2a and b). For δ_H (Figure S. 2c), while a slightly lower value (*ca.* 7.40 $MPa^{1/2}$) appears under conditions where δ_P ranges from 12 $MPa^{1/2}$ to 13 $MPa^{1/2}$ and δ_H equals to 0 $MPa^{1/2}$, the region is found in what you might term a hypothetical area, where no known solvents can be found. Outside this hypothetical scenario, the prediction converges consistently to δ_H values between *ca.* 7.8 $MPa^{1/2}$ and 8.0 $MPa^{1/2}$, closely aligning with the *MLoc*-predicted minima of δ_H (7.96 $MPa^{1/2}$) while *MLoc* effectively avoids predicting HSPs in the impractical solvent regions. Additionally, the convergence speed, indicated by the number of iteration steps (Figure S. 2d), shows that grid search requires approximately 160 to 1,800 steps to converge in this test, with six cases (marked as red crosses) failing to converge within the maximum iteration steps (10,000). In contrast, *MLoc* requires only 254 steps.

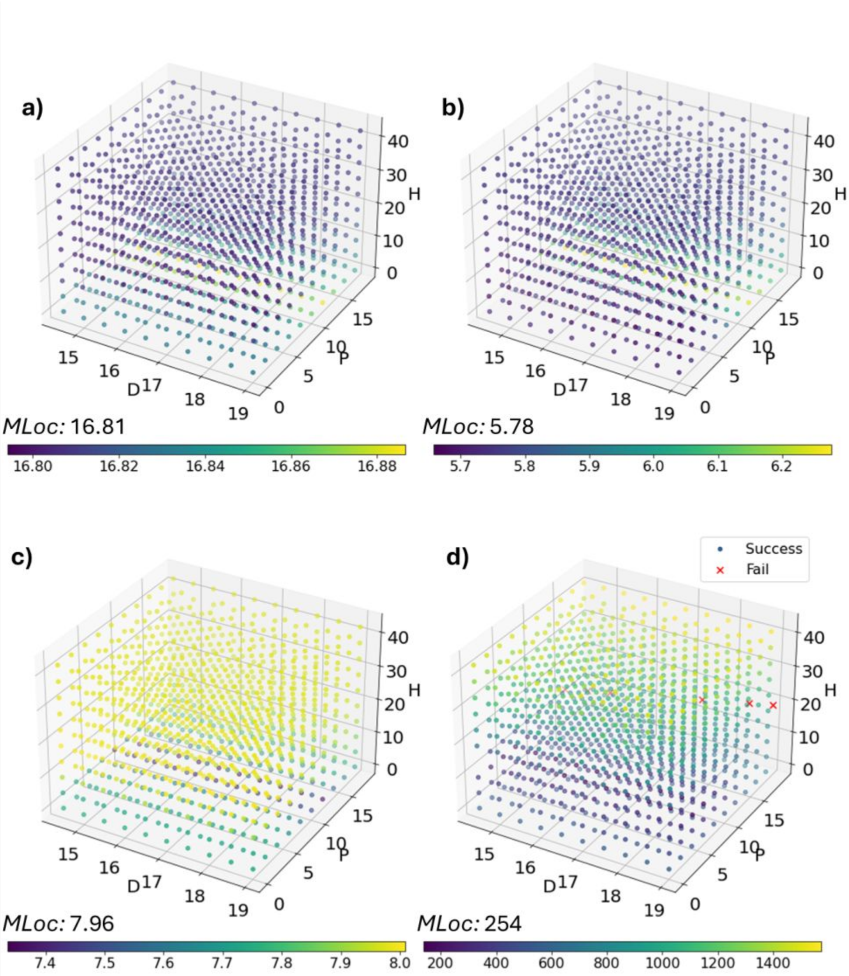

Figure S. 2 Comparison of the outcome of MLoc algorithm with a grid-search method to represent the outcome of using random guess to initiate the optimization of a) δ_D, b) δ_P, and c) δ_H, with total iteration steps shown in d). MLoc effectively predicted the minima for both δ_D and δ_P, and avoided reaching impractical solvent regions as otherwise risked by grid search in the case of δ_H. The total iteration steps used by MLoc are only 254 steps, whereas the random guess may range from 160 to 1800 steps, at a risk of failing to converge at certain starting points (labelled as red crosses) within 10,000 iterations.

1.8. Explanation of the conceptual similarity and differences of *MLoc* with k-means

The optimization process of the centroid location process of a k-means clustering algorithm is an inspiration of the *MLoc* algorithm, despite the target application of *MLoc* is distinguished from an often multi-cluster focused k-means algorithm.

While k-means and *MLoc* share a conceptual similarity in that a distance between each data point in a known dataset and an unknown centroid is utilized to optimize and confirm the location of the centroid, the distance in the two contexts is different: *MLoc* takes the Hansen distance instead of

Euclidean distance used in k-means, where Hansen distance is distorted by two folds in the dispersion dimension as shown in Eq. 3.[a]

Additionally, *MLoc* focuses on providing a physical-meaningful optimization outcome rather than taking a geometry average if otherwise adopted in a single-cluster k-means scenario.[b] The centroid optimization process in the *MLoc* algorithm incorporates both the Hansen solubility theory and Beer–Lambert law. These two objectives are optimized simultaneously here to keep the material as close to a good solvent as possible in the Hansen space, and for the UV/Vis absorbance of the dispersed material in the best solvent to be as high as possible. To account for both criteria, a simple weighted average is challenging to formulate analytically, hence requiring advanced optimization methods as developed here.

1.9. Proof of the convexity of the optimization function

We have considered the convexity of the optimization function in Eq. 4, which can be proved using the Jensen's inequality theorem in this problem:

The properties of a convex function include that an affine map and a non-negative weighted sum of a convex function preserve convexity.

In this problem, we can prove that

1) Hansen distances can be represented as an affine map of Euclidean norm, which is known to be a convex function, and

2) the function to be optimized in Eq. 4 in the manuscript, reproduced below, is a non-negative weighted sum of a set of Hansen distances, given UV/Vis absorption values are all non-negative numbers.

$$\underset{\boldsymbol{\delta}_M}{argmin} \sum_{i=1}^{n} w_i R_i\,(\boldsymbol{\delta}_M)$$

Therefore, the optimization function in Eq. 4 only exists as a single, global minimum.

To prove the existence of the affine map, consider a Euclidean norm of any given HSP:

$$\|\boldsymbol{\delta}\| = \sqrt{\delta_D^2 + \delta_P^2 + \delta_H^2} \qquad \textit{Eq. S. 7}$$

The Hansen distance can be expressed as an affine transform of a Euclidean norm:

---

[a] Hansen in the HSP user guidebook has explained that this is because the dipolar and hydrogen-bonding interactions are shared by a pair of molecules, whereas the dispersion term is all contributed to by atomic force. Therefore, focusing on an individual molecule, δ_P and δ_H only share half contribution compared to δ_D, *i.e.*, a ½ coefficient is expected before the δ_P and δ_H terms, which is normalized as a factor of 2 before the δ_D term for clarity.[40]

[b] In a one-cluster scenario, k-means is equivalent to simply taking the average of all data points. Transferring this approach to HSPs, this would mean taking the average of HSPs of all test solvents. The predicted HSPs will then be independent of the properties of a material and solely depends on the selection of test solvents.

$$R_i(\boldsymbol{\delta}_M) = \sqrt{4\left(\delta_D^{(M)} - \delta_D^{(i)}\right)^2 + \left(\delta_P^{(M)} - \delta_P^{(i)}\right)^2 + \left(\delta_H^{(M)} - \delta_H^{(i)}\right)^2} \quad \textit{Eq. S. 8}$$

$$= \|\boldsymbol{A}\boldsymbol{\delta}_M - \boldsymbol{b}\|, \quad \textit{Eq. S. 9}$$

where

$$\boldsymbol{A} = \begin{bmatrix} 2 & 0 & 0 \\ 0 & 1 & 0 \\ 0 & 0 & 1 \end{bmatrix}, \quad \textit{Eq. S. 10}$$

$$\boldsymbol{b} = \begin{bmatrix} 2\delta_D^{(i)} \\ \delta_P^{(i)} \\ \delta_H^{(i)} \end{bmatrix}. \quad \textit{Eq. S. 11}$$

This represents the affine map between Hansen distance and Euclidean distance.

2. Supporting information for *MLoc*

The *MLoc* workflow was developed using Python 3. Dependencies of *MLoc* include matplotlib,[47] pandas,[48] seaborn[49] and numpy.[50] This workflow has been tested on a personal computer with Microsoft Windows 10 as the operating system, a 3.20 GHz CPU (Intel Core i7-8700 processor), and 16 GB random-access memory (RAM). The algorithm normally completes within seconds with negligible computing cost (*ca.* 100 MB memory).

Explanation of each feature of the built-in database is available in Table S. 1 in section S.2.1 below. The detailed procedure for sample preparation, UV/Vis measurement and input document preparation are introduced in S.2.2, S.2.3. The procedure to load *MLoc* input files is introduced in S.2.4. Hyperparameters for gradient descent, including the learning rate, maximum number of iteration cycles and tolerance of convergence, have been set by default. All hyperparameters are adjustable while necessary (S.2.5). Output settings are available in S.2.6. Procedures to run the *MLoc* main algorithm are illustrated in S.2.7. Anticipated outcomes are described in S. 2.8. Common troubleshooting advice is provided in S.2.9.

2.1. Built-in database

The structure of the built-in database is shown in Scheme S. 3.

No.	CAS	Name	D	P	H	Mole_vol	ims_idx
1	75-07-0	Acetaldehyde	14.7	12.5	7.9	56.6	
2	64-19-7	Acetic acid	14.5	8	13.5	57.1	139;190;39
3	108-24-7	Acetic anhydride	16	11.7	10.2	94.5	40
4	67-64-1	Acetone	15.5	10.4	7	74	
5	75-05-8	Acetonitrile	15.3	18	6.1	52.6	139;47;136;240;190
6	98-86-2	Acetophenone	19.6	8.6	3.7	117.4	243
7	107-13-1	Acrylonitrile	16	12.8	6.8	67.1	243
8	107-18-6	Allyl alcohol	16.2	10.8	16.8	68.4	
9	628-63-7	Amyl acetate	15.8	3.3	6.1	148	243
10	62-53-3	Aniline	19.4	5.1	10.2	91.5	
11	100-66-3	Anisole	17.8	4.1	6.7	119.1	243
12	100-52-7	Benzaldehyde	19.4	7.4	5.3	101.5	
13	71-43-2	Benzene	18.4	0	2	89.4	243;14;27;69;74;104;116;120;135;214;220;233
14	108-46-3	1,3-Benzenediol	18	8.4	21	87.5	13;44;120
15	65-85-0	Benzoic acid	18.2	6.9	9.8	113.1	243

bp	mw	viscosity	vis_temp	heat_of_vap	hov_temp	SMILES	alias	synonyms
20.8	44.05	0.21	20	25.73	20.2	O=CC		acetic aldehyde;ethyl aldehyde
117	60.05	1.06	25	23.7	117.9	CC(=O)O		ethanoic acid;Ethylic acid;Vinegar acid
139	102.09	0.84	25	38.2	139.5	CC(=O)OC(=O)C		Acetyl acetate;Acetanhydride;Ethanoic anhydride
56.2	58.08	0.32	20	29.1	56.05	CC(=O)C		2-propanone
81.6	41.05	0.35	20	29.81	80	CC#N	ACN	Methyl cyanide;Cyanomethane
202	120.15	1.68	-1	49	-1	CC(=O)C1=CC=CC=C1		1-Phenylethanone;Methyl phenyl ketone;Acetylbenzene
77.2	53.06	0.34	25	32.6	25	C=CC#N		2-Propenenitrile;Polyacrylonitrile;Prop-2-enenitrile
96.9	58.08	1.22	25	40	97.4	C=CCO		2-Propen-1-ol;Vinylcarbinol
149.2	130.18	1.58	11	38.42	149.2	CCCCCOC(=O)C		Pentyl acetate;n-Amyl acetate
184	93.13	4.35	20	55.83	-1	C1=CC=C(C=C1)N		Benzenamine;Phenylamine;Aminobenzene
155.5	108.14	0.78	30	46.84	25	COC1=CC=CC=C1		Methoxybenzene;Methyl phenyl ether
178.7	106.12	1.32	25	42.5	179	C1=CC=C(C=C1)C=O		Benzoic aldehyde;Phenylmethanal
80.08	78.11	0.6	25	33.83	25	C1=CC=CC=C1		benzol

Scheme S. 3 A snapshot of the MLoc built-in database.

Features included in the current database are listed and explained in Table S. 1. Data were collected from *PubChem* and the HSPs user guidebook. Any entry labelled with "-1" or "None" indicates that data were not available.

Table S. 1 Explanation of the built-in database. All data provided are experimentally reported values.

Feature	Explanation
No.	No. of solvent in this database
CAS	CAS No. of corresponding solvent
Name	Solvent name
D	Dispersion term (δ_D), unit: MPa$^{1/2}$
P	Dipolar term (δ_P), unit: MPa$^{1/2}$
H	Hydrogen bond term (δ_H), unit: MPa$^{1/2}$
Mole_vol	Molar volume, unit: cm^3 mol^{-1}
ims_idx	Index of immiscible entries in this database
bp	Boiling point, unit: °C
mw	Molecular weight, unit: g mol^{-1}
viscosity	Viscosity of corresponding solvent, unit: Pa s
vis_temp	Temperature at which the viscosity is measured, unit: °C
heat_of_vap	Molar heat of evaporation, unit: kJ mol^{-1}
hov_temp	Temperature at which the heat_of_vap is measured, unit: °C
SMILES	Simplified molecular-input line-entry system of corresponding solvent
alias	Common abbreviations of corresponding solvent
synonyms	Common synonyms of corresponding solvent

The database can be edited by users if necessary. If multiple entries need to be stored under one category, for example, synonyms, each entry needs to be separated by a semicolon, WITHOUT a space left in-between. Commas, spaces, and hyphens are allowed to be left in the entry name.

Example: name_1;name_2;name with space;name-with-hyphen;name, with, comma;name_5

2.2. Preparation of polymer suspensions and UV/Vis data

To prepare input UV/Vis data (solubility indicators) for *MLoc*, aliquots of polymer suspensions in a series of solvents need to be prepared according to the following steps (Scheme S. 4):

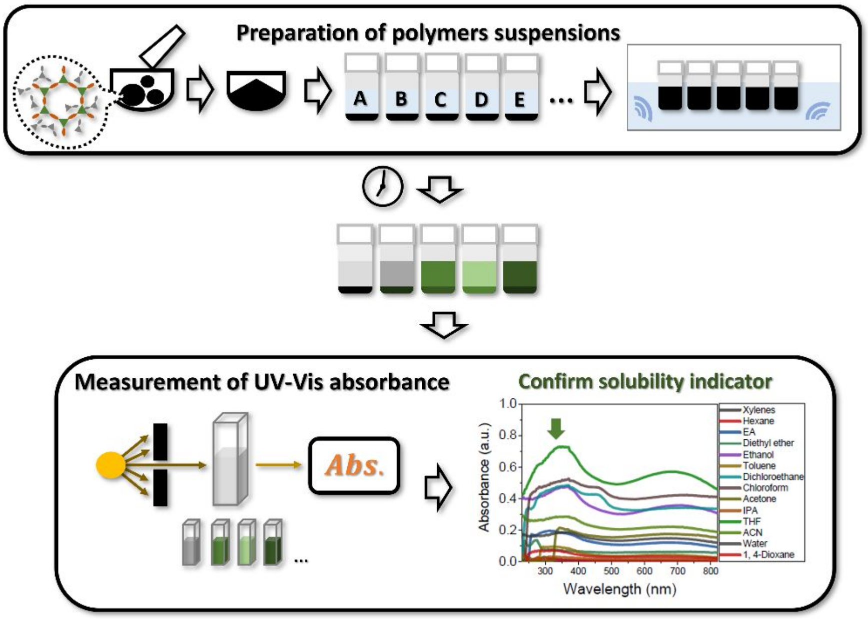

Scheme S. 4 Procedure for obtaining experimental absorbance data of polymer suspensions.

1) Grind the polymers to a fine powder using mortar and pestle (or a bench-top ball mill). 2) Disperse equal amounts of polymer powder (5 mg each) into a series of different solvents of interest (5 mL each). 3) We recommend using a series of solvents to cover a broad range in the Hansen space (*i.e.*, each of the partial HSPs should be widely spread across this solvent set). In the tests of this workflow, the number of tested solvents was between 10 to 15. To support the selection of test solvents, we provide an example list of solvents that are broadly distributed in Hansen space (Table S. 2). This list is available in the *MLoc* repository: *example_solvent.csv*.[51] More options (249 solvents) can be found in the built-in solvent database, *db.csv*.

Table S. 2 A list of common lab solvent candidates and corresponding HSPs.

CAS	Solvent	δ_D/MPa$^{1/2}$	δ_P /MPa$^{1/2}$	δ_H /MPa$^{1/2}$
110-54-3	Hexane	14.9	0.0	0.0
1330-20-7	Xylene	17.6	1.0	3.1

108-88-3	Toluene	18.0	1.4	2.0
123-91-1	1,4-Dioxane	19.0	1.8	7.4
60-29-7	Diethyl ether	14.5	2.9	5.1
67-66-3	Chloroform	17.8	3.1	5.7
141-78-6	Ethyl acetate	15.8	5.3	7.2
109-99-9	Tetrahydrofuran	16.8	5.7	8.0
67-63-0	Isopropanol	15.8	6.1	16.4
107-06-2	1,2-Dichloroethane	19.0	7.4	4.1
64-17-5	Ethanol	15.8	8.8	19.4
67-64-1	Acetone	15.5	10.4	7.0
127-19-5	*N,N*-Dimethylacetamide	16.8	11.5	10.2
872-50-4	1-Methyl-2-pyrrolidone	18.0	12.3	7.2
68-12-2	Dimethylformamide	17.4	13.7	11.3
616-47-7	1-Methylimidazole	19.7	15.6	11.2
7732-18-5	Water	15.5	16.0	42.3
67-68-5	Dimethyl sulfoxide	18.4	16.4	10.2
75-05-8	Acetonitrile	15.3	18.0	6.1

4) Tightly seal the sample vial with parafilm. 5) Sonicate the suspensions for a minimum of 2 h in a standard laboratory sonication bath. 6) After sonication, allow the suspension to settle for 2 h before further analysis. 7) Pipette out an appropriate volume of the supernatant from the vial and record the UV-Vis absorbance spectrum of each solution. Baseline correction needs to be done with the neat solvent used in the polymer suspension. 8) After recording the full UV-Vis absorbance spectrum (from 220 nm to 800 nm), pick the maximum characteristic absorbance of each sample as the solubility indicator. If multiple absorption bands exist, the maximum characteristic peak needs to be selected in the region contributed to by the same spectroscopic transition mechanism for each solvent. For UV-responsive systems, we recommend using the maximum absorbance originating from the polymer sample's benzenoid band (below 400 nm) owing to better resolution.

2.3. Preparing an input document for solvent candidates

The input experimental data should be based on solvent-dependent properties associated with the concentration of solute in saturated solutions. The program has been tested using UV/Vis absorbance of polymer suspensions. However, we recommend that users consider additional characterization methods where necessary. Input data needs to be provided in *input_mloc_data.csv*[52] before loading the main *MLoc* program.

To prepare the input document of solvent candidates, open *db.csv* in the directory where *MLoc* is installed. Search solvent name or alias to get corresponding CAS No. Open *input_mloc_data.csv* in the

directory where *MLoc* is installed. Fill in the CAS No. and solvent name in the "CAS" and "Solvent" columns, respectively. Then fill in the absorbance data in the "Indicator" column. For example, if the maximum characteristic absorbance of the studied polymer in tetrahydrofuran (THF) is 0.7, in acetonitrile (ACN) is 0.5, the corresponding input would be Table S. 3. Save *input_mloc_data.csv* and close the file.

Table S. 3 Example input format.

CAS	Solvent	Indicator
109-99-9	THF	0.7
75-05-8	Acetonitrile	0.5

The CAS No. of solvents used in experiments can be obtained from standard chemistry databases, for example, PubChem,[53] or the built-in database of *MLoc* (*db.csv).*[54] We recommend searching for the CAS No. from the built-in database and copying that into the input file. Using a wrong CAS No. will cause wrong HSPs to be entered for the rest of these calculations and will affect the accuracy of the results. Make sure special characters are avoided in the "CAS" column, for example, spaces, ".", or "/". Do not omit "0" or "-" in the CAS No. Unexpected formatting can lead to program error.

"Indicator" must be a number. Using the wrong data type can lead to an error.

There is no restriction for the "Solvent" column. Solvent names do not affect calculation parameters but will appear on the output spreadsheet and figures.

While preparing the candidate list, do not input repeated entries or leave blank rows or edit the title row.

2.4. Loading input documents

Install Python and *MLoc*. We recommend Python is installed from anaconda[55]:

https://docs.anaconda.com/free/anaconda/install/

Open *MLoc_main.py* using any code editor. Check the setup block (Scheme S. 5).

```
import MLoc_io
import MLoc_fetch_info
import MLoc_calc
from datetime import datetime
from datetime import date

#Setup block - please edit if necessary

# name of database and input data
db_name, usr_input_data = 'db.csv', 'input_mloc_data.csv'

# hyperparameters for gradient descent
alpha = 0.01
n_max = 1000000
tol = 0.005

# prefix of output files
op_name_prefix = 'test'


# Main program below
db, usr_input = MLoc_io.read_input_csv(db_name, usr_input_data)
```

Scheme S. 5 Setup block of MLoc_main (row 9 to 18), highlighted in the red frame.

Go to the first part of the setup block. Edit the input documents line with corresponding file names for database (*db_name*, default '*db.csv*') and input data (*usr_input_data*, default '*input_mloc_data.csv*'), if different from those provided. Save *MLoc_main.py* once editing is completed.

```
db_name, usr_input_data = 'db.csv', 'input_mloc_data.csv'
```

Make sure the correct files are loaded and the file extension (.csv) is entered. Both files must be stored in the same working directory as the main program. If a user renames these two files, for example,

'*MyName_db.csv*', '*MyPolymer_today.csv*', then the above input row needs to be updated as:

```
db_name, usr_input_data = 'MyName_db.csv', 'MyPolymer_today.csv'
```

2.5. Setting hyperparameters for gradient descent

Go to the second part of the setup block (Scheme S. 5), which corresponds to hyperparameters for gradient descent. Edit the learning rate (*alpha*, default 0.01), maximum iteration steps (*n_max*, default 1,000,000), and tolerance of convergence (*tol*, default 0.005), if necessary. Save *MLoc_main.py* once editing is completed. Note that we recommend initially using the default setup for this step unless convergence fails.

2.6. Setting output filename

Go to the final part of the setup block (Scheme S. 5) to customize the output file name (*op_name_prefix*, default '*test*'). The name set here is the prefix of the output file. Save *MLoc_main.py* once editing is completed.

2.7. Running *MLoc*

With all the input data ready and setup completed, run the main *MLoc* program in the terminal using the following command:

```
python MLoc_main.py
```

The program will first fetch the HSPs from the database. If this step has completed successfully, the following message will be shown in the terminal:

```
Fetching HSP done.
```

If successful, the main calculation step is carried out in the next step. Scheme S. 6 is an output example in the user terminal if the calculation is successful.

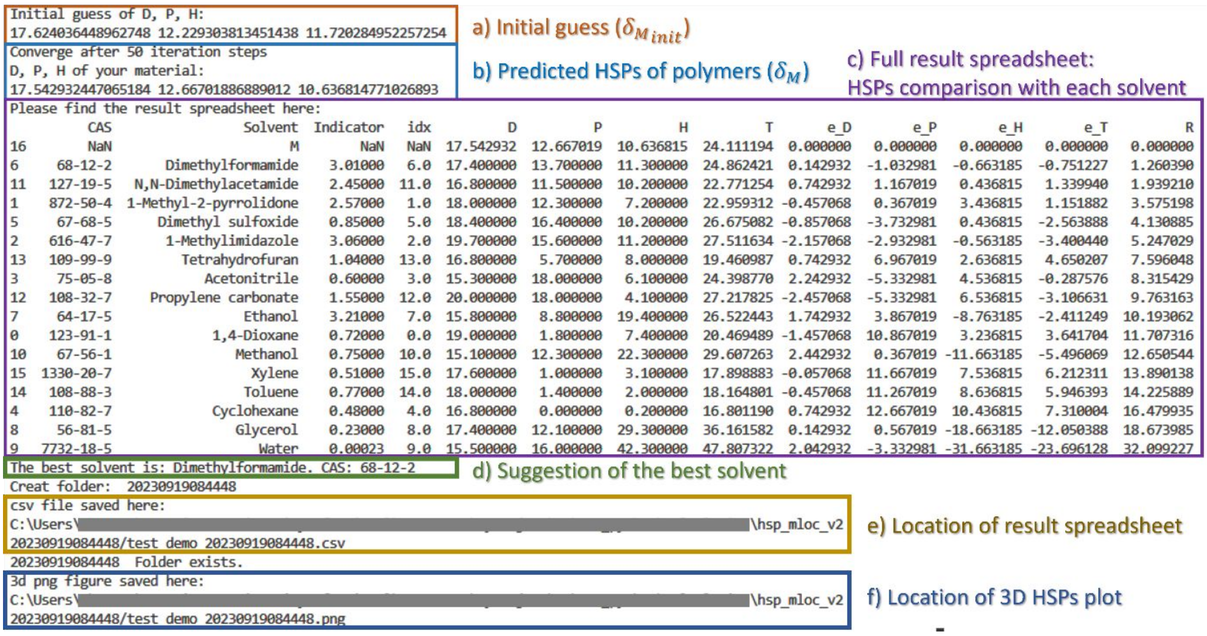


```
Initial guess of D, P, H:
17.624036448962748 12.229303813451438 11.720284952257254
Converge after 50 iteration steps
D, P, H of your material:
17.542932447065184 12.66701886889012 10.636814771026893
Please find the result spreadsheet here:
         CAS                 Solvent  Indicator   idx          D          P          H          T        e_D        e_P        e_H        e_T          R
16       NaN                       M        NaN   NaN  17.542932  12.667019  10.636815  24.111194   0.000000   0.000000   0.000000   0.000000   0.000000
6    68-12-2       Dimethylformamide    3.01000   6.0  17.400000  13.700000  11.300000  24.862421   0.142932  -1.032981  -0.663185  -0.751227   1.260390
11  127-19-5   N,N-Dimethylacetamide    2.45000  11.0  16.800000  11.500000  10.200000  22.771254   0.742932   1.167019   0.436815   1.339940   1.939210
1   872-50-4  1-Methyl-2-pyrrolidone    2.57000   1.0  18.000000  12.300000   7.200000  22.959312  -0.457068   0.367019   3.436815   1.151882   3.575198
5    67-68-5      Dimethyl sulfoxide    0.85000   5.0  18.400000  16.400000  10.200000  26.675082  -0.857068  -3.732981   0.436815  -2.563888   4.130885
2   616-47-7      1-Methylimidazole     3.06000   2.0  19.700000  15.600000  11.200000  27.511634  -2.157068  -2.932981  -0.563185  -3.400440   5.247029
13  109-99-9         Tetrahydrofuran    1.04000  13.0  16.800000   5.700000   8.000000  19.460987   0.742932   6.967019   2.636815   4.650207   7.596048
3    75-05-8            Acetonitrile    0.60000   3.0  15.300000  18.000000   6.100000  24.398770   2.242932  -5.332981   4.536815  -0.287576   8.315429
12  108-32-7     Propylene carbonate    1.55000  12.0  20.000000  18.000000   4.100000  27.217825  -2.457068  -5.332981   6.536815  -3.106631   9.763163
7    64-17-5                 Ethanol    3.21000   7.0  15.800000   8.800000  19.400000  26.522443   1.742932   3.867019  -8.763185  -2.411249  10.193062
0   123-91-1             1,4-Dioxane    0.72000   0.0  19.000000   1.800000   7.400000  20.469489  -1.457068  10.867019   3.236815   3.641704  11.707316
10   67-56-1                Methanol    0.75000  10.0  15.100000  12.300000  22.300000  29.607263   2.442932   0.367019 -11.663185  -5.496069  12.650544
15 1330-20-7                  Xylene    0.51000  15.0  17.600000   1.000000   3.100000  17.898883  -0.057068  11.667019   7.536815   6.212311  13.890138
14  108-88-3                 Toluene    0.77000  14.0  18.000000   1.400000   2.000000  18.164801  -0.457068  11.267019   8.636815   5.946393  14.225889
4   110-82-7             Cyclohexane    0.48000   4.0  16.800000   0.000000   0.200000  16.801190   0.742932  12.667019  10.436815   7.310004  16.479935
8    56-81-5                Glycerol    0.23000   8.0  17.400000  12.100000  29.300000  36.161582   0.142932   0.567019 -18.663185 -12.050388  18.673985
9  7732-18-5                   Water    0.00023   9.0  15.500000  16.000000  42.300000  47.807322   2.042932  -3.332981 -31.663185 -23.696128  32.099227
The best solvent is: Dimethylformamide. CAS: 68-12-2
Creat folder:  20230919084448
csv file saved here:
C:\Users\[redacted]\hsp_mloc_v2
20230919084448/test demo 20230919084448.csv
20230919084448  Folder exists.
3d png figure saved here:
C:\Users\[redacted]\hsp_mloc_v2
20230919084448/test demo 20230919084448.png
```

Scheme S. 6 Example output in the terminal. a) Initial guess (Row 1 and 2). b) Calculation details and predicted HSPs of the target polymers (Row 3 to 5). c) Full result spreadsheet. Solvents are ranked by the difference in Hansen distance (the R column). Details in difference of each partial HSP are presented in the "e_D, P, H, T" columns. d) Suggestion of the best solvent. e) The location of the result spreadsheet. f) The location of the 3D HSPs plot.

The initial guess is generated according to Eq. 7 and displays in the terminal window (Scheme S. 6a). The target HSPs (δ_M) are calculated using a gradient-descent approach. If the calculation has completed, a message to confirm this, indicating the iteration steps and the result, will present to the user (Scheme S. 6b). A detailed results spreadsheet, comparing HSPs for polymers and each solvent is generated (Scheme S. 6c). Solvents are sorted in ascending order by the Hansen distance (*R*), with the best one identified as the closest to the polymer (Scheme S. 6d). A copy of this spreadsheet is saved in a folder named with the current time. The location of saved results is indicated on the terminal (Scheme S. 6e). A 3D plot of all tested solvents and the target polymer in the Hansen space (Scheme S. 7) are stored in the same folder. The figure and its location are presented to the user (Scheme S. 6f).

Where personalization of the 3D plot is required, the plot setup can be edited in the "*plot_hsp*" function in *MLoc_io.py* using standard commands of *matplotlib*.[47]

2.8. Anticipated outcomes

The first row in the results spreadsheet shows the predicted HSPs of the studied polymer ($\boldsymbol{\delta_M}$). The remaining rows rank all the tested solvents based on the Hansen distance from the polymer. The input absorbance information is stored in the "Indicator" column. Each solvent is assigned with an ID in the "idx" column. The ID corresponds to the annotation of data points in the output plot of the Hansen space (Figure 2). HSPs and total solubility parameters of all solvents are listed in the next four columns ("D", "P", "H", "T"). Each difference term between solvents and polymers ($\Delta\delta_D$, $\Delta\delta_P$ and $\Delta\delta_H$) is summarized in the "e_D", "e_P", "e_H" and "e_T" columns, respectively. The final column "R" represents the Hansen distance of each solvent candidate.

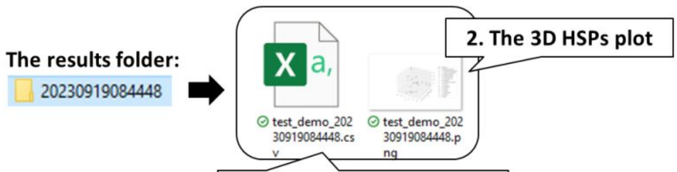

1. The results spreadsheet

1.

	Predicted result	Input	ID	δ_D	δ_P	δ_H	δ_T	$\Delta\delta_D$	$\Delta\delta_P$	$\Delta\delta_H$	$\Delta\delta_T$	Hansen distance
CAS	Solvent	Indicator	idx	D	P	H	T	e_D	e_P	e_H	e_T	R
	M			17.5429	12.667	10.6368	24.1112	0	0	0	0	0
68-12-2	Dimethylformamide	3.01	6	17.4	13.7	11.3	24.8624	0.14293	-1.03298	-0.66319	-0.75123	1.26039
127-19-5	N,N-Dimethylacetamide	2.45	11	16.8	11.5	10.2	22.7713	0.74293	1.16702	0.43681	1.33994	1.93921
872-50-4	1-Methyl-2-pyrrolidone	2.57	1	18	12.3	7.2	22.9593	-0.45707	0.36702	3.43681	1.15188	3.5752
67-68-5	Dimethyl sulfoxide	0.85	5	18.4	16.4	10.2	26.6751	-0.85707	-3.73298	0.43681	-2.56389	4.13089
616-47-7	1-Methylimidazole	3.06	2	19.7	15.6	11.2	27.5116	-2.15707	-2.93298	-0.56319	-3.40044	5.24703
109-99-9	Tetrahydrofuran	1.04	13	16.8	5.7	8	19.461	0.74293	6.96702	2.63681	4.65021	7.59605
75-05-8	Acetonitrile	0.6	3	15.3	18	6.1	24.3988	2.24293	-5.33298	4.53681	-0.28758	8.31543
108-32-7	Propylene carbonate	1.55	12	20	18	4.1	27.2178	-2.45707	-5.33298	6.53681	-3.10663	9.76316
64-17-5	Ethanol	3.21	7	15.8	8.8	19.4	26.5224	1.74293	3.86702	-8.76319	-2.41125	10.1931
123-91-1	1,4-Dioxane	0.72	0	19	1.8	7.4	20.4695	-1.45707	10.867	3.23681	3.6417	11.7073
67-56-1	Methanol	0.75	10	15.1	12.3	22.3	29.6073	2.44293	0.36702	-11.6632	-5.49607	12.6505
1330-20-7	Xylene	0.51	15	17.6	1	3.1	17.8989	-0.05707	11.667	7.53681	6.21231	13.8901
108-88-3	Toluene	0.77	14	18	1.4	2	18.1648	-0.45707	11.267	8.63681	5.94639	14.2259
110-82-7	Cyclohexane	0.48	4	16.8	0	0.2	16.8012	0.74293	12.667	10.4368	7.31	16.4799
56-81-5	Glycerol	0.23	8	17.4	12.1	29.3	36.1616	0.14293	0.56702	-18.6632	-12.0504	18.674
7732-18-5	Water	0.00023	9	15.5	16	42.3	47.8073	2.04293	-3.33298	-31.6632	-23.6961	32.0992

2.

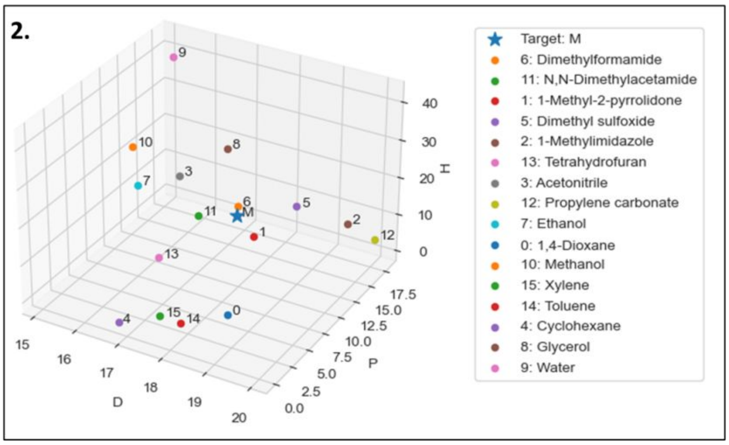

Scheme S. 7 *Explanation of MLoc output. A new folder is created and named according to the time when the calculation was performed, when a result spreadsheet (1) and a 3D HSPs plot (2) were created. The spreadsheet file contains key calculation results. The highlighted block in the red frame refers to the calculated HSPs of the target material. Columns, from left to right, correspond to: CAS No. (CAS), solvent name (Solvent), input indicator value (Indicator), solvent ID during the calculation (ID), dispersion (δ_D), dipolar (δ_P), hydrogen bond (δ_H) partial HSPs, Hildebrand parameter (δ_T), difference in the dispersion ($\Delta\delta_D$), dipolar ($\Delta\delta_P$), hydrogen bond ($\Delta\delta_H$) HSPs, the Hildebrand parameter ($\Delta\delta_T$) between target material and the solvent candidate, and the Hansen distance (R).*

2.9. Troubleshooting

A list of potential problems and their solutions is summarized in Table S. 4 and Scheme S. 8.

Table S. 4 Troubleshooting of common problems.

Problem	Potential cause	Solution
Failed to load input files. "FileNotFoundError" is raised. (Scheme S. 8a)	The file name of the database and input experimental data is not correctly entered, or a document is not saved in the same working directory as the main program.	Check the input files are stored in the same directory as *MLoc_main.py.* Make sure the file name is correctly entered, without missing the extension (*.csv*).
MLoc raises a value error saying "CAS Not Found". The error CAS No. is indicated in the terminal (Scheme S. 8b).	The CAS No. of certain solvents in the input document is not available in the database, or the wrong CAS No. has been entered.<br>The format of "CAS" is wrong. "0" or "-" is missing or misplaced in "CAS" column. For example, 75-05-8 is misinput as 75-5-8 or 75-0-58.	Check the input document: Make sure the correct CAS No. has been entered. Avoid any special characters.
MLoc raises a value error saying "Indicator is not a number" (Scheme S. 8c).	The data put in the "Indicator" column in the input document includes special characters or text.	Check the input document and check the correct number has been entered in the "Indicator" column. Avoid any special characters or text.
A warning message says "Reached maximum iterations. Fail to converge" (Scheme S. 8d).	Certain input data have an abnormally high value compared to the rest of the data.<br>Learning rate is too low or convergence tolerance is too strict.	Check the input "Indicator" is correct: No data with abnormally high values (for example, larger than 10). Absorbance of polymer supernatant is not expected to be over 10. Double check experimental steps if in doubt.<br><br>If the quality of data have been checked, carefully, increase the learning rate (*alpha*) by 0.01 or the tolerance (*tol*) by 0.005.<br><br>We don't recommend increasing the maximum iteration step (*n_max*), as this will reduce the efficiency of this algorithm.

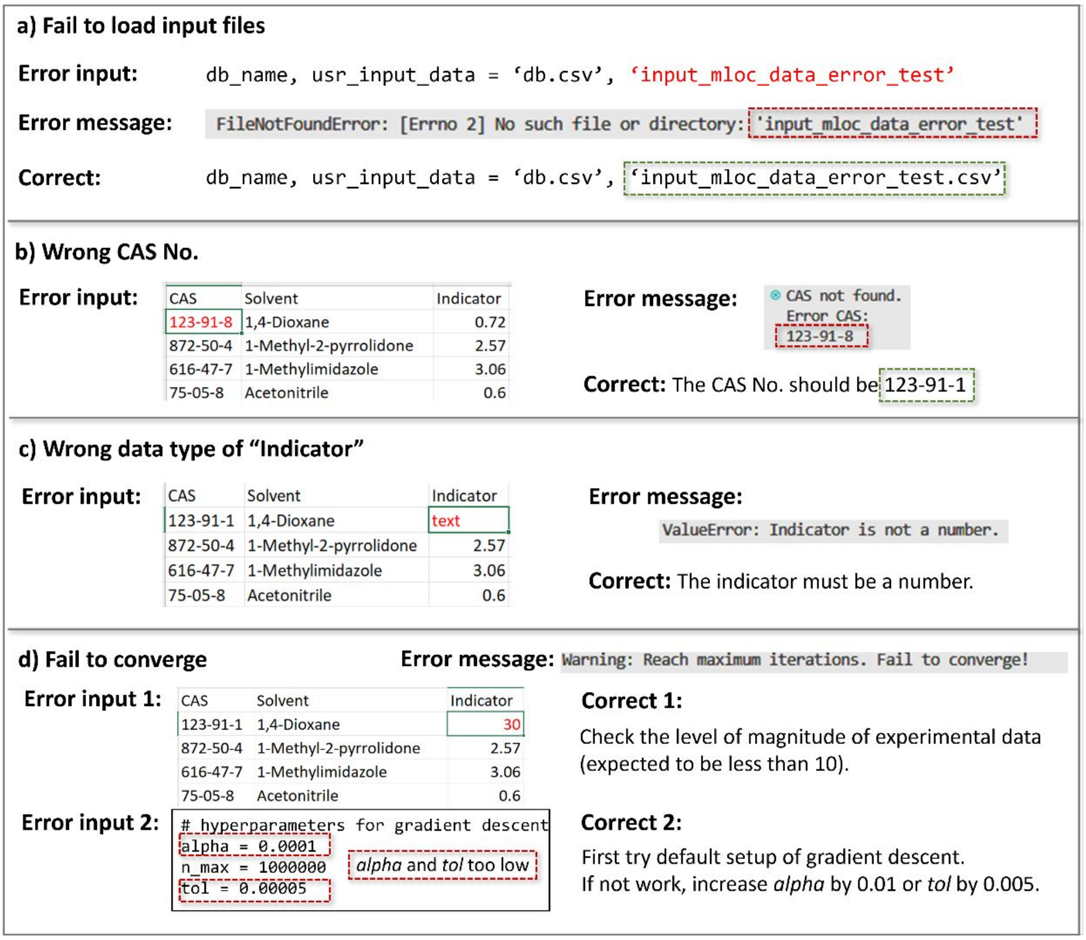

Scheme S. 8 Example troubleshooting cases. a) Failed to load input files. b) Wrong CAS No. was reported. c) Wrong data type in the “Indicator” column was detected. d) Failed to converge.

3. Supporting information for experimental work

3.1. Chemicals

All reagents were purchased from commercial suppliers and utilized as received.

Table S. 5 Suppliers of chemicals (and solvents) used in the case study experiments.

Name	Source
1,4-Dioxane	Alfa Aesar
2-Dicyclohexylphosphino-2’,4’,6’-triisopropylbiphenyl	Sigma-Aldrich
2,5-Dibromobenzoic acid	Alfa Aesar
Barium sulfate	Wako
Bis(dibenzylideneacetone)palladium(0)	Sigma-Aldrich
Chloroform	Sigma-Aldrich
Methanol	Sigma-Aldrich

p-Phenylenediamine	Alfa Aesar
Sodium tert-butoxide	Alfa Aesar
Tetrahydrofuran (for synthesis)	Anhydrous Engineering (University of Bristol)
Toluene (for synthesis)	Anhydrous Engineering (University of Bristol)
Tris(4-bromophenyl)amine	Santa Cruz Biotechnology
Hexane	Sigma-Aldrich
Ethyl acetate	Sigma-Aldrich
Acetonitrile	Fisher Chemical
Xylene (for UV/Vis measurement)	Fisher Chemical
Toluene (for UV/Vis measurement)	Fisher Chemical
Ethanol	Sigma-Aldrich
Diethyl ether	Sigma-Aldrich
1,2-Dichloroethane	Sigma-Aldrich
Acetone	Sigma-Aldrich
Isopropanol	Sigma-Aldrich
Tetrahydrofuran (for UV/Vis measurement)	Sigma-Aldrich

3.2. Instrumental details and methodologies for characterization

Fourier-transform infrared (FTIR) spectra of samples were obtained on a PerkinElmer Spectrometer from 4000 cm^{-1} to 450 cm^{-1}.

Ultraviolet-visible-near infrared (UV-vis-NIR) spectra were determined using a Shimadzu spectrophotometer from 220 nm to 1400 nm (barium sulphate was utilized as the background for solid state measurement).

Powder X-ray diffraction (PXRD) measurements were conducted using a Bruker D8 Advance instrument equipped with a PSD LynxEye detector and a copper (Cu) Kα radiation source (λ = 1.54 Å). Diffraction patterns were collected in the 2θ range of 5–60° with a step size of 0.02° and an exposure time of 1 s $step^{-1}$. Samples were dried and ground to achieve an approximate homogenous particle size before analysis. A silicon sample holder in flat plate geometry was used for each sample.

Thermogravimetric analysis (TGA) was performed using a TGA Q500 at a ramp rate of 10 °C min^{-1} under an N_2 atmosphere.

Scanning electron microscopy (SEM) images were recorded on a JEOL JSM-IT300 microscope. A thin layer of silver (Ag, 99.99% purity), typically 15–20 nm, was coated onto the samples in argon (Ar), to prevent electron charging, using a high-resolution sputter coater from Agar Scientific. Micrographs were obtained on a JEOL JSM-IT300, operated at an accelerating voltage of 15 kV at a working distance of approximately 10 mm, detecting secondary and backscattered electrons.

Gas sorption measurements of N_2 at 77 K and CO_2 at 273 K and 298 K were collected using a Quantachrome Autosorb iQ instrument. Before sorption studies, the samples were dried on the Schlenk line at 150 °C and then degassed. The degassing procedure was carried out under a high vacuum in three stages:

- Heating of the sample to 50 ˚C at 1 ˚C/min followed by holding for 20 min;
- Heating of the sample to 100 ˚C at 2 ˚C/min followed by holding for 100 min;
- Heating of the sample to 180 ˚C at 2 ˚C/min followed by holding for 500 min.

3.3. PTPA163 synthesis and characterization.

3.3.1. *Synthesis*

The synthesis of **PTPA163** is adapted from a reported method,[56] illustrated in Scheme S. 8.

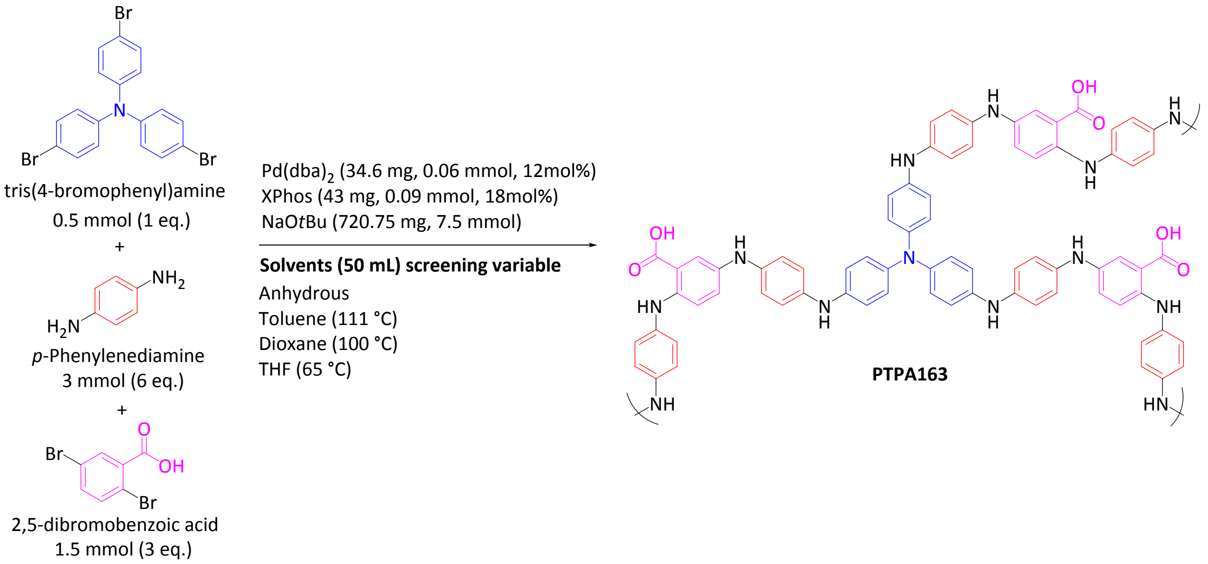

*Scheme S. 8 Synthesis of **PTPA163** in different solvents.*

PTPA163 synthesized in toluene, THF and 1, 4-dioxane are denoted PTPA-tol-1:6:3, PTPA-THF-1:6:3 and PTPA-dxn-1:6:3 below, respectively.

PTPA163 was characterized using FTIR spectroscopy, UV/Vis spectroscopy, PXRD, TGA, and SEM. Gas sorption measurements of N_2 at 77 K and CO_2 at 273 K were performed on Quantachrome Autosorb iQ instrument. Thorough degassing was performed prior to gas adsorption measurements to ensure no residual solvent remained in the samples. The specific surface areas were calculated by the BET method in the relative pressure (P/P_0) range from 0.05 to 0.30 at 77 K. The total pore volumes were measured at a relative pressure of $P/P_0 \approx 1$. The pore size distributions (PSD) were derived from the adsorption branches of the N_2 isotherms using the nonlocal density functional theory (NLDFT) equilibrium model at 77 K, assuming slit pore geometry on carbon.[57]

3.3.2. *Component characterization: FTIR*

FTIR spectra of **PTPA163** synthesized at different temperatures using different solvents (Figure S. 3) provide proof of successful synthesis. However, changes in these factors slightly affected the chemical structure. The peaks for the C–Br group of tris(4-bromophenylamine) at 1178 cm^{-1} and for the $–NH_2$ group from *p*-phenylenediamine at 3420 cm^{-1} are strongly reduced compared with the starting materials (Figure S. 4), which confirms the formation of these materials.

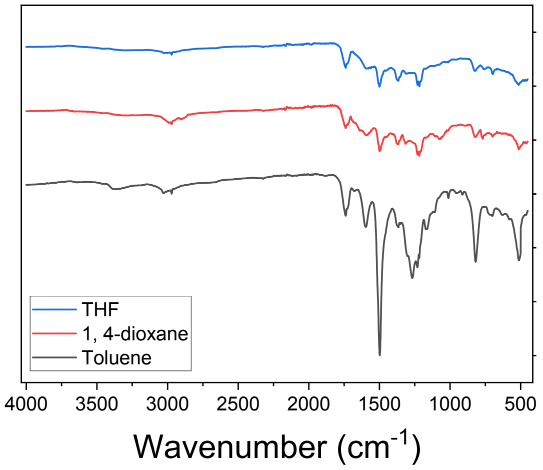

*Figure S. 3 FTIR spectra of **PTPA163** synthesized in toluene, 1, 4-dioxane, and THF.*

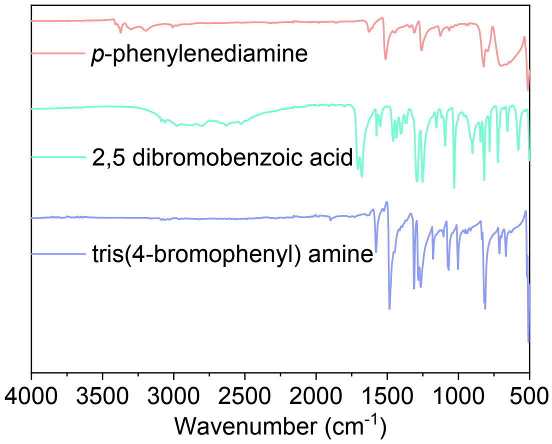

*Figure S. 4 FTIR spectra of all starting materials for **PTPA163**.*

3.3.3. Component characterization: solid-state UV-Vis-NIR

For all materials, the solid-state UV-vis-NIR spectra (Figure S. 5) show a broad peak (~710 nm) and a narrow peak (~370 nm), which are ascribed to the π-π* transition of quinoid and benzenoid rings respectively.

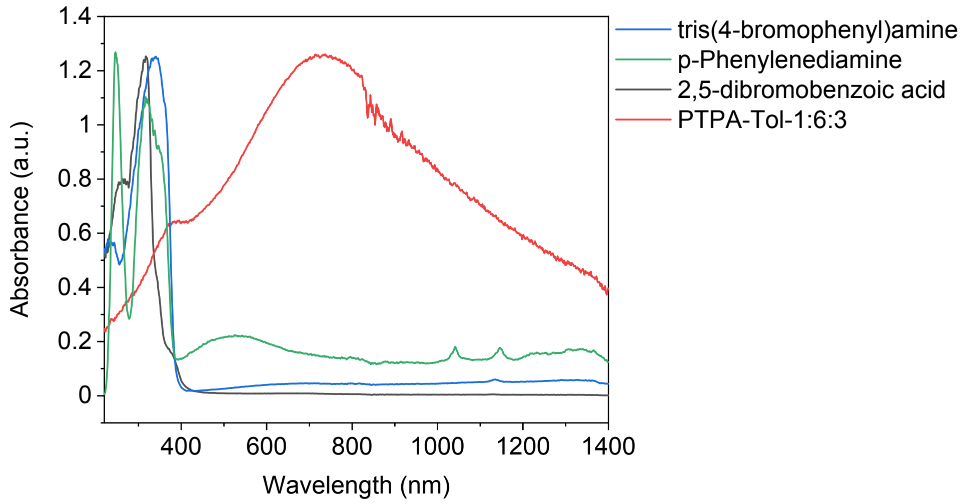

Figure S. 5 Solid-state UV-vis-NIR spectra of starting materials tris(4-bromophenyl)amine, p-phenylenediamine, 2,5-dibromobenzoic acid and as-prepared PTPA-Tol-1:6:3.

3.3.4. Morphology characterization: SEM

SEM images show the morphology of materials synthesized in toluene, dioxane and THF (Figure S. 6). A similar amorphous morphology was detected in either of the studied solvents. All polymers show high degrees of aggregation producing interstitial voids with consequent non-uniform porosity.

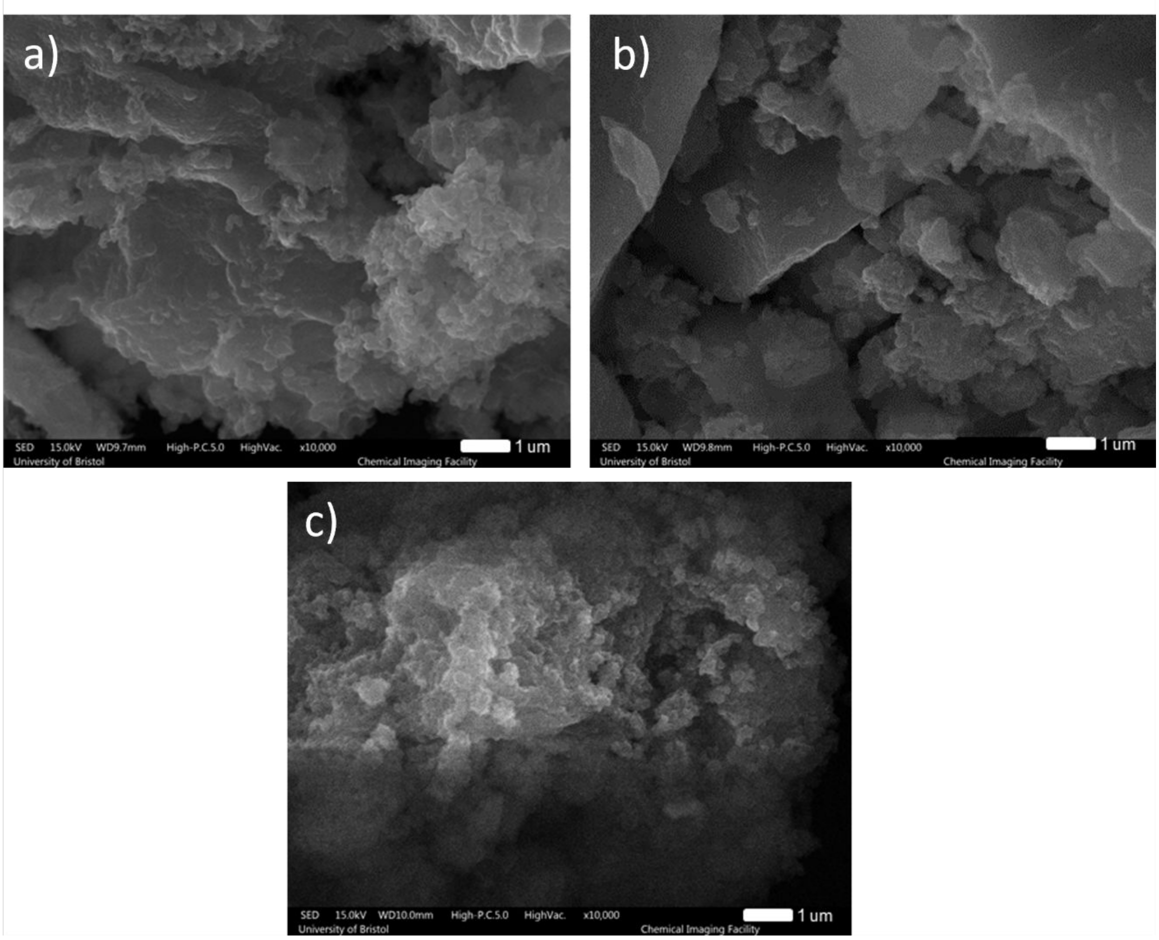

Figure S. 6 SEM images of a) PTPA-Tol-1:6:3; b) PTPA-Dxn-1:6:3, and c) PTPA-THF-1:6:3 networks.

3.3.5. Morphology characterization: PXRD

PXRD analysis (Figure S. 6) revealed the absence of sharp Bragg diffraction peaks, indicating the successful removal of precursors and catalysts as well as confirming the amorphous nature of the polymeric material. These findings are consistent with the FTIR results (Figure S. 3), indicating the absence of starting materials. Furthermore, the absence of a π-stacking peak at approximately 2θ = 26° corroborates the amorphous structure of **PTPA163**. The broad diffraction feature observed at 2θ ≈ 12° is attributed to the weak scattering from the amorphous silicon (Si) sample holder (part number: C79298A3244B249).

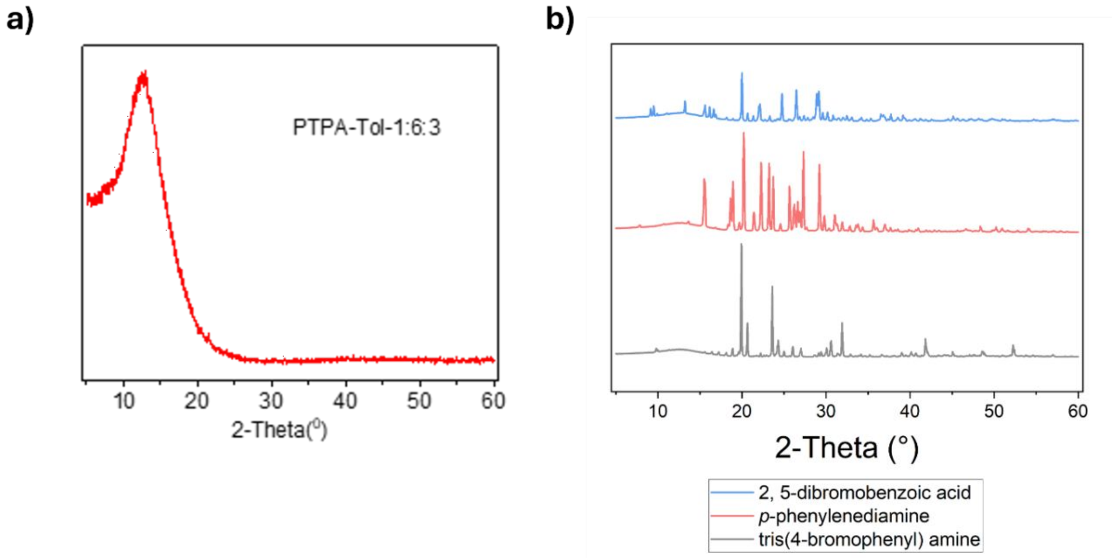

Figure S. 7 Powder XRD patterns of a) ***PTPA163*** *and of b) all starting materials.*

3.3.6. Stability characterization: TGA

TGA was performed to assess the thermal stability of the materials. **PTPA163** is thermally stable in N_2 up to 250 °C (Figure S. 8). The stability is mainly owing to the abundance of cross-linking within the polymeric network. The sample gradually loses mass with > 70% of the initial mass remaining at 800 °C.

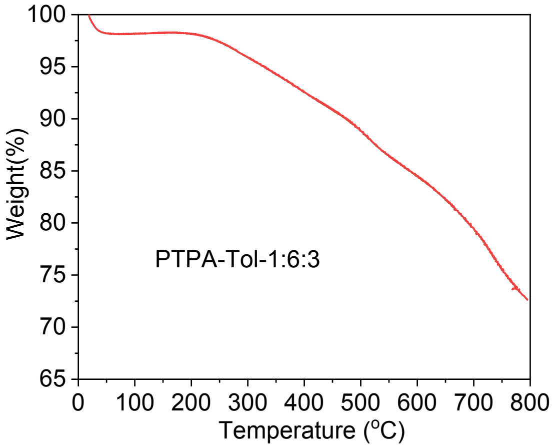

Figure S. 8 TG curve of PTPA163.

3.3.7. Summary of the average synthesis outcome and characterization of porosity

The average BET surface area, micropore volume, total pore volume, CO_2 uptake and yield of **PTPA163** synthesized in toluene, 1, 4-dioxane and THF, are provided in Table S. 6. The C-constant from the BET fitting plots presented in Section 3.3.9 is also provided in this table.

Table S. 6 Synthesis and porosity characterization summary of PTPA163.

Polymer	S_{BET} (m^2 g^{-1})$^{a)}$	Micropore volume (cm^3 g^{-1})$^{b)}$	Total pore volume (cm^3 g^{-1})	CO_2 uptake at 1 bar, 273 K (wt%)	C parameter from BET fitting	Average Yield (%)
PTPA-THF-1:6:3	357±7	0.082	0.360	6.95±0.35	112	5
PTPA-Dxn-1:6:3	271±6	0.042	0.101	4.86±0.24	80	5
PTPA-Tol-1:6:3	108±4	0.019	0.214	3.44±0.17	71	37

[a)] Calculated from N_2 adsorption isotherms collected at 77 K using the BET method (the uncertainty of N_2 uptake is calculated by the standard deviation over two to three repeated experiments); [b)] Cumulative pore volume in pore sizes ≤ 2 nm calculated from N_2 adsorption isotherms collected at 77 K using NLDFT. *Values may fall in the error of the measurement and should be treated with caution. Note that the low-yield groups have been found over repeated experiments and are hard to get good yield at corresponding conditions.

It needs to be noted that this work focuses on the optimization of CO_2 uptake. In the field of porous materials, there is only a limited amount of evidence showing clear correlation among BET surface area, pore volume and CO_2 uptake.[58] According to the data presented in Table S. 6, we note that the changes in CO_2 uptake are not strictly correlated to the BET surface area, micropore volume, or total pore volume. BET characterization is based on the use of nitrogen molecules, which differ significantly from CO_2 molecules in terms of molecular geometry, kinetic diameter, and other physicochemical properties. These differences can therefore influence how the materials interact with CO_2.

*3.3.8. Pore size distribution (PSD) characterization of **PTPA163**.*

The pore size distribution of **PTPA163** in toluene, dioxane and THF is presented in Figure S. 9.

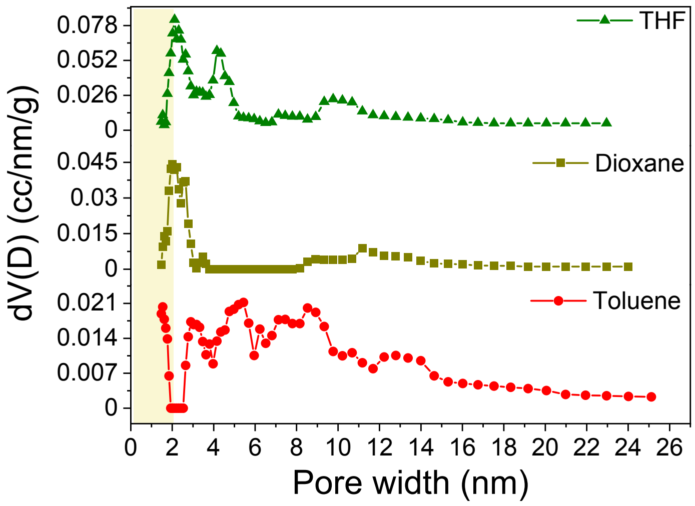

*Figure S. 9 PSD plots of **PTPA163** synthesized in toluene, 1,4-dioxane, and THF.*

PTPA163 synthesized from THF (the optimum solvent predicted by *MLoc*) resulted in a hierarchical pore size distribution. The majority of pores locates in the range from 2 to 7 nm in diameter, with two peaks centered at 3 nm and 6 nm. A small fraction of pores, ranging from 12 to 15 nm in diameter, are distributed in the meso-porous range.

PTPA163 synthesized in dioxane was less porous than that generated by synthesis in THF, in terms of the total pore volume. Approximately half of the pores are located in the 2 to 5 nm region. The other half are spread over the region from 9 to 20 nm.

The system in toluene demonstrates a more uniform pore size distribution compared with both THF and dioxane. The pore size covers a wide range, from 3 to 20 nm.

The result confirmed that better solvent predicted by *MLoc* can efficiently control the pore size distribution.

*3.3.9. BET isotherms and linear plots of **PTPA163***

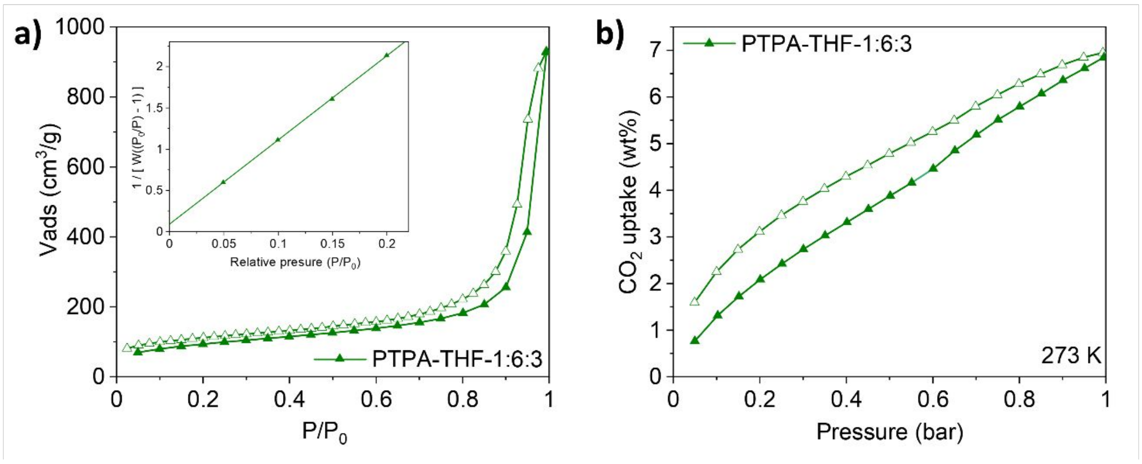

*Figure S. 10 a) N_2 sorption isotherm collected at 77 K and multipoint BET plot and b) CO_2 sorption isotherm collected at 273 K of **PTPA163** synthesized from THF. Adsorption branches represented by filled symbols and desorption branches by empty symbols.*

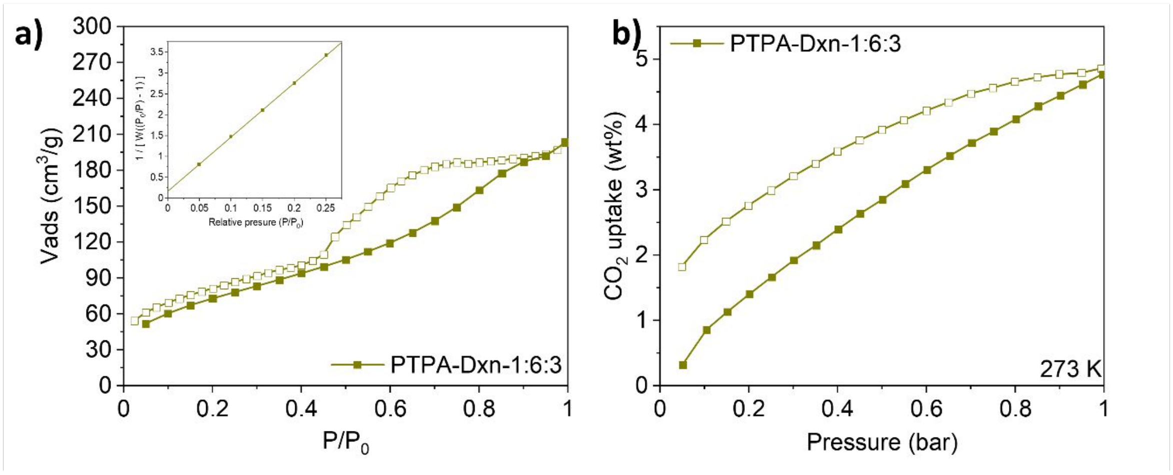

*Figure S. 11 a) N_2 sorption isotherm collected at 77 K and multipoint BET plot and b) CO_2 sorption isotherm collected at 273 K of **PTPA163** synthesized from 1,4-dioxane. Adsorption branches represented by filled symbols and desorption branches by empty symbols.*

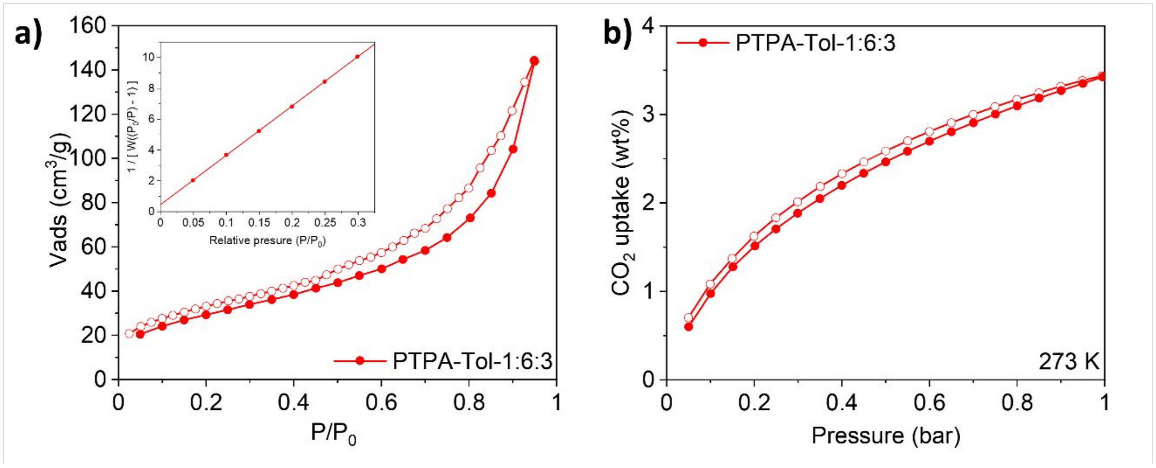

*Figure S. 12 a) N_2 sorption isotherm collected at 77 K and multipoint BET plot and b) CO_2 sorption isotherm collected at 273 K of **PTPA163** synthesized from toluene. Adsorption branches represented by filled symbols and desorption branches by empty symbols.*

3.4. Discussion of the contribution of partial HSPs for PTPA163

While acknowledging the complexity of solvent impact, we note from the 1, 4-dioxane entry in Table 2 in the manuscript that the dispersion term is contributing to a lesser extent than the dipolar and hydrogen-bonding terms. This follows intuitively that in a molecular that contains polar functional groups the dispersion interaction, representing the non-polar contribution of molecular interactions, is typically weaker than the dipole–dipole interaction and the hydrogen-bonding interaction.

3.5. Full data for PTPA163 predicted by *MLoc*

The full input data of for **PTPA163** is provided in the main manuscript, in Table 1.

The full output data for **PTPA163** is shown in Scheme S. 9 and Figure S. 13. Note: the data addressed in the case study takes the absolute difference between solvents and M. The user output includes the sign of each difference.

CAS	Solvent	Indicator	idx	D	P	H	T	e_D	e_P	e_H	e_T	R
	M			16.81	5.78	7.96	19.48	0.00	0.00	0.00	0.00	0.00
109-99-9	Tetrahydrofuran	0.7283	12	16.80	5.70	8.00	19.46	0.01	0.08	-0.04	0.02	0.09
141-78-6	Ethyl acetate	0.19783	1	15.80	5.30	7.20	18.15	1.01	0.48	0.76	1.33	2.21
67-66-3	Chloroform	0.52733	9	17.80	3.10	5.70	18.95	-0.99	2.68	2.26	0.53	4.03
67-64-1	Acetone	0.21496	10	15.50	10.40	7.00	19.94	1.31	-4.62	0.96	-0.45	5.39
123-91-1	1,4-Dioxane	0.07471	6	19.00	1.80	7.40	20.47	-2.19	3.98	0.56	-0.99	5.95
107-06-2	1,2-Dichloroethane	0.48543	8	19.00	7.40	4.10	20.80	-2.19	-1.62	3.86	-1.32	6.06
60-29-7	Diethyl ether	0.13713	7	14.50	2.90	5.10	15.64	2.31	2.88	2.86	3.84	6.15
1330-20-7	Xylene	0.03041	3	17.60	1.00	3.10	17.90	-0.79	4.78	4.86	1.58	7.00
108-88-3	Toluene	0.09716	4	18.00	1.40	2.00	18.16	-1.19	4.38	5.96	1.32	7.78
67-63-0	Isopropanol	0.03261	11	15.80	6.10	16.40	23.58	1.01	-0.32	-8.44	-4.10	8.68
110-54-3	Hexane	0.02821	0	14.90	0.00	0.00	14.90	1.91	5.78	7.96	4.58	10.56
64-17-5	Ethanol	0.47671	5	15.80	8.80	19.40	26.52	1.01	-3.02	-11.44	-7.04	12.00
75-05-8	Acetonitrile	0.28546	2	15.30	18.00	6.10	24.40	1.51	-12.22	1.86	-4.92	12.72
7732-18-5	Water	0.18804	13	15.50	16.00	42.30	47.81	1.31	-10.22	-34.34	-28.33	35.92

Scheme S. 9 Full calculation details of PTPA163 (hyperparameters used default setup).

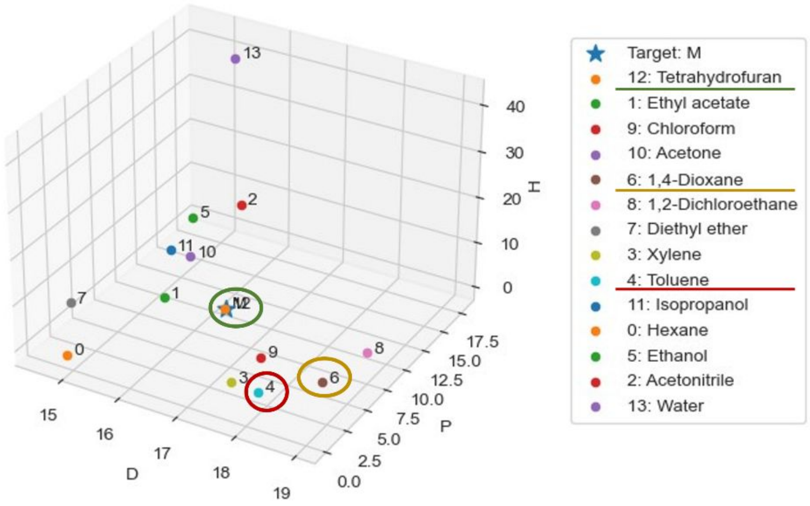

Figure S. 13 Original output figure of PTPA163, with three solvents of interest highlighted.

3.6. Experimental details and UV/Vis data for POPs in the HSP-POP database

The experimental details for the synthesis of POPs in the HSP–POP database are provided in this section. Group 7 is the main case study discussed in the manuscript. The synthesis details are provided in ESI-3.3.1. Group 1 to 6 and Group 12 to 15 follow the same protocol as Group 7. Group 8 to 11 were prepared according to the procedures reported in 2014.[59] Group 16 follows a polycondensation mechanism that differs from Group 1 to 15 (B–H coupling reaction) according to the method reported earlier.[44]

Solvent abbreviations used in this section are provided in Table S. 7.

Table S. 7 Solvent abbreviations used in the training set for HSP-POPs databases.

Solvent	Abbreviation
N-methyl-2-pyrrolidone	NMP
N-methylimidazole	NMI
Dichloromethane	DCM
Dimethylformamide	DMF
Dimethylsulfoxide	DMSO

3.6.1. Group 1: PTPA1O

A Schlenk tube was charged with tris(4-bromophenyl) amine (0.50 mmol, 241 mg), 4,4'-ethylenedianiline (0.75 mmol, 159 mg) to obtain the 1:1.5 ratio of core to linker. Pd(dba)$_2$ (0.03 mmol, 17.3 mg, 4 mol%), XPhos (0.045 mmol, 22 mg), NaOtBu (3.50 mmol, 336 mg) was placed under a nitrogen atmosphere. Anhydrous toluene (30 mL) was added, and the reaction mixture was heated

with stirring to 111 °C. After 48 h, the mixture was cooled to room temperature and the products were then washed with chloroform, ethanol, and methanol (200 mL each) to remove the catalyst, salt and oligomers, followed by a Soxhlet extraction with methanol and chloroform, respectively for 24 h each. The product was dried in a vacuum oven to yield corresponding amine networks as powder.

Table S. 8 Input information (UV/Vis absorbance) of PTPA1O (Group 1).

CAS	Solvent	Indicator (UV/Vis absorbance at λ_{max})
110-54-3	Hexane	0.22
141-78-6	Ethyl acetate	0.03
64-17-5	Ethanol	0.04
108-88-3	Toluene	0.26
75-09-2	DCM	0.90
67-66-3	Chloroform	1.09
67-64-1	Acetone	0.20
67-63-0	Isopropanol	0.09
109-99-9	Tetrahydrofuran	0.06
75-05-08	Acetonitrile	0.03
7732-18-5	Water	0.00
123-91-1	1,4-Dioxane	0.03
67-68-5	DMSO	0.71
1330-20-7	Xylene	0.48

3.6.2. Group 2: PTPA-2O

A Schlenk tube was charged with tris(4-bromophenyl) amine (0.50 mmol, 241 mg), 4,4'-stilbenediamine (0.75 mmol, 158 mg) to obtain the 1:1.5 ratio of core to linker. $Pd(dba)_2$ (0.03 mmol, 17 mg, 4 mol%), XPhos (0.045 mmol, 22 mg), NaOtBu (3.50 mmol, 336 mg) was placed under a nitrogen atmosphere. Anhydrous toluene (30 mL) was added, and the reaction mixture was heated with stirring to 111 °C. After 48 h, the mixture was cooled to room temperature and the products were then washed with chloroform, ethanol, and methanol (200 mL each) to remove the catalyst, salt and oligomers, followed by a Soxhlet extraction with methanol and chloroform, respectively for 24 h each. The product was dried in a vacuum oven to yield corresponding amine networks as powder.

Table S. 9 Input information (UV/Vis absorbance) of PTPA-2O (Group 2).

CAS	Solvent	Indicator (UV/Vis absorbance at λ_{max})
110-54-3	Hexane	0.01
141-78-6	Ethyl acetate	0.65
64-17-5	Ethanol	0.02
108-88-3	Toluene	0.12
75-09-2	DCM	1.03
67-66-3	Chloroform	1.19
67-64-1	Acetone	0.65
67-63-0	Isopropanol	1.10
109-99-9	Tetrahydrofuran	1.12
75-05-08	Acetonitrile	0.61

7732-18-5	Water	0.00
123-91-1	1,4-Dioxane	0.75
67-68-5	DMSO	1.09
1330-20-7	Xylene	0.14

3.6.3. Group 3: PTPA-3O

A Schlenk tube was charged with tris(4-bromophenyl) amine (0.50 mmol, 241 mg), *p*-phenylenediamine (0.75 mmol, 81 mg) to obtain the 1:1.5 ratio of core to linker. $Pd(dba)_2$ (0.03 mmol, 17 mg, 4 mol%), XPhos (0.045 mmol, 22 mg), NaOtBu (3.50 mmol, 336 mg) was placed under a nitrogen atmosphere. Anhydrous toluene (30 mL) was added, and the reaction mixture was heated with stirring to 111 °C. After 48 h, the mixture was cooled to room temperature and the products were then washed with chloroform, ethanol, and methanol (200 mL each) to remove the catalyst, salt and oligomers, followed by a Soxhlet extraction with methanol and chloroform, respectively for 24 h each. The product was then dried in a vacuum oven to yield corresponding amine networks as powder.

Table S. 10 Input information (UV/Vis absorbance) of PTPA-3O (Group 3).

CAS	Solvent	Indicator (UV/Vis absorbance at λ_{max})
110-54-3	Hexane	0.07
141-78-6	Ethyl acetate	0.70
64-17-5	Ethanol	0.83
108-88-3	Toluene	0.12
75-09-2	DCM	0.65
67-66-3	Chloroform	1.02
67-64-1	Acetone	0.83
67-63-0	Isopropanol	0.97
109-99-9	Tetrahydrofuran	0.11
75-05-08	Acetonitrile	0.42
7732-18-5	Water	0.52
123-91-1	1,4-Dioxane	0.17
67-68-5	DMSO	0.93
1330-20-7	Xylene	0.09

3.6.4. Group 4: PTPA-4O

Schlenk tube was charged with tris(4-bromophenyl) amine (0.50 mmol, 241 mg) as core, 4,4`-diaminodiphenyl sulfide (0.75 mmol, 162 mg) to obtain the 1:1.5 ratio of core to linker. $Pd(dba)_2$ (0.03 mmol, 17 mg, 4 mol%), XPhos (0.045 mmol, 22 mg), NaOtBu (3.50 mmol, 336 mg) was placed under a nitrogen atmosphere. Anhydrous toluene (30 mL) was added, and the reaction mixture was heated with stirring to 111 °C. After 48 h, the mixture was cooled to room temperature and the products were then washed with chloroform, ethanol, and methanol (200 mL each) to remove the catalyst, salt and oligomers, followed by a Soxhlet extraction with methanol and chloroform, respectively for 24 h each. The product was dried in a vacuum oven to yield corresponding amine networks as powder.

Table S. 12 Input information (UV/Vis absorbance) of PTPA-4O (Group 4).

CAS	Solvent	Indicator (UV/Vis absorbance at λ_{max})
110-54-3	Hexane	0.02
141-78-6	Ethyl acetate	0.86
64-17-5	Ethanol	0.32
108-88-3	Toluene	0.21
75-09-2	DCM	0.91
67-66-3	Chloroform	0.95
67-64-1	Acetone	0.63
67-63-0	Isopropanol	0.26
109-99-9	Tetrahydrofuran	0.79
75-05-08	Acetonitrile	0.19
7732-18-5	Water	0.03
123-91-1	1,4-Dioxane	0.42
67-68-5	DMSO	0.80
1330-20-7	Xylene	0.24

3.6.5. Group 5 (PTPA-4O-U1A1) and 6 (PTPA-4O-U1B1)

A Schlenk tube was charged with tris(4-bromophenyl) amine (0.50 mmol, 241 mg) as core, 4,4`-diaminodiphenyl sulfide and 1,4-dibromobenzene to obtain a 1:6:3 ratio (for Group 5) and a 2:5:2 ratio (for Group 6). Pd(dba)$_2$ (0.03 mmol, 17 mg), XPhos (0.045 mmol, 22 mg), NaOtBu (3.50 mmol, 336 mg) was placed under a nitrogen atmosphere. Anhydrous toluene (30 mL) was added, and the reaction mixture was heated with stirring to 111°C. After 48 h, the mixture was cooled to room temperature and the products were then washed with chloroform, ethanol, and methanol (200 mL each) to remove the catalyst, salt and oligomers, followed by a Soxhlet extraction with methanol and chloroform, respectively for 24 h each. The product was dried in a vacuum oven to yield corresponding amine networks as powder.

Table S. 12 Input information (UV/Vis absorbance) of PTPA-4O-U1A1 (Group 5).

CAS	Solvent	Indicator (UV/Vis absorbance at λ_{max})
110-54-3	Hexane	0.01
141-78-6	Ethyl acetate	0.60
64-17-5	Ethanol	0.42
108-88-3	Toluene	0.24
75-09-2	DCM	0.34
67-66-3	Chloroform	0.48
67-64-1	Acetone	0.77
67-63-0	Isopropanol	0.56
109-99-9	Tetrahydrofuran	1.08
75-05-08	Acetonitrile	0.12
7732-18-5	Water	0.00
123-91-1	1,4-Dioxane	1.01
67-68-5	DMSO	1.07
1330-20-7	Xylene	0.23

Table S. 13 Input information (UV/Vis absorbance) of PTPA-4O-U1B1 (Group 6).

CAS	Solvent	Indicator (UV/Vis absorbance at λ_{max})

110-54-3	Hexane	0.003
141-78-6	Ethyl acetate	0.13
64-17-5	Ethanol	0.04
108-88-3	Toluene	0.10
75-09-2	DCM	0.26
67-66-3	Chloroform	0.58
67-64-1	Acetone	0.11
67-63-0	Isopropanol	0.09
109-99-9	Tetrahydrofuran	0.18
75-05-08	Acetonitrile	0.04
7732-18-5	Water	0.00
123-91-1	1,4-Dioxane	0.17
67-68-5	DMSO	0.92
1330-20-7	Xylene	0.17

3.6.6. Group 8: CMP-TP-1

A Schlenk tube was charged with tris(4-bromophenyl)amine (0.90 mmol, 434 mg) as core, 4'-(4-bromophenyl)-2,2':6',2''-terpyridine (BPTP) (0.10 mmol, 39 mg) as co-monomer, and *p*-phenylenediamine (1.50 mmol, 162 mg) as linker to obtain a 9:1:15 ratio. $Pd(dba)_2$ (17.3 mg, 0.03 mmol) 2-dicyclohexylphosphino-2',4',6'-triisopropylbiphenyl (XPhos, 0.045 mmol, 22 mg), and sodium tert-butoxide (NaOtBu, 2 mmol, 192 mg) was added and placed under a nitrogen atmosphere. Anhydrous toluene (50 mL) was added, and the reaction mixture was heated with stirring to 110°C. After 48 h, the reaction was cooled to room temperature and solvents were then removed by centrifugation. The remaining solids were washed with chloroform, hot deionized water, and methanol (200 mL each) to remove the catalyst, salt and oligomers, followed by Soxhlet extraction with methanol and chloroform, respectively for 24 h. The product was then dried in a vacuum oven to yield corresponding amine networks as powder.

Table S. 14 Input information (UV/Vis absorbance) of CMP-TP-1 (Group 8).

CAS	Solvent	Indicator (UV/Vis absorbance at λ_{max})
67-68-5	DMSO	1.16
68-12-2	DMF	0.82
75-05-08	Acetonitrile	0.26
872-50-4	NMP	0.99
108-32-7	Propylene carbonate	0.70
1330-20-7	Xylene	0.03
108-88-3	Toluene	0.10
64-17-5	Ethanol	0.56
123-91-1	Dioxane	0.06
67-66-3	Chloroform	0.30
67-64-1	Acetone	0.14
75-09-2	Dichloromethane	0.31
141-78-6	Ethyl acetate	0.13
110-54-3	Hexane	0.00
109-99-9	THF	0.35

7732-18-5	Water	0.00

3.6.7. Group 9: CMP-TP-4

A Schlenk tube was charged with tris(4-bromophenyl)amine (0.60 mmol, 289 mg) as core, 4'-(4-bromophenyl)-2,2':6',2''-terpyridine (BPTP) (0.40 mmol, 155 mg) as co-monomer, and *p*-phenylenediamine (1.50 mmol, 162 mg) as linker to obtain a 6:4:15 ratio. $Pd(dba)_2$ (0.03 mmol, 17 mg) 2-dicyclohexylphosphino-2',4',6'-triisopropylbiphenyl (XPhos, 0.045 mmol, 22 mg), and sodium tert-butoxide (NaOtBu, 2 mmol, 192 mg) was added and placed under a nitrogen atmosphere. Anhydrous toluene (50 mL) was added, and the reaction mixture was heated with stirring to 110°C. After 48 h, the reaction was cooled to room temperature and solvents were then removed by centrifugation. The remaining solids were washed with chloroform, hot deionized water, and methanol (200 mL each) to remove the catalyst, salt and oligomers, followed by Soxhlet extraction with methanol and chloroform, respectively for 24 h each. The product was then dried in a vacuum oven to yield corresponding amine networks as powder.

Table S. 15 Input information (UV/Vis absorbance) of CMP-TP-4 (Group 9).

CAS	Solvent	Indicator (UV/Vis absorbance at λ_{max})
67-68-5	DMSO	1.16
68-12-2	DMF	0.97
75-05-08	Acetonitrile	0.77
872-50-4	NMP	1.04
108-32-7	Propylene carbonate	0.45
1330-20-7	Xylene	0.003
108-88-3	Toluene	0.05
64-17-5	Ethanol	0.78
123-91-1	Dioxane	0.18
67-66-3	Chloroform	0.62
67-64-1	Acetone	0.11
75-09-2	Dichloromethane	0.24
141-78-6	Ethyl acetate	0.05
110-54-3	Hexane	0.00
109-99-9	THF	0.42
7732-18-5	Water	0.0

3.6.8. Group 10: CMP-BP-1

A Schlenk tube was charged with tris(4-bromophenyl)amine (0.90 mmol, 434 mg) as core, 4,4'-dibromo-2,2'-bipyridine (DBBP) (0.10 mmol, 32 mg) as co-monomer, and *p*-phenylenediamine (PPD) (1.5 mmol, 162 mg) as linker to obtain a 9:1:15 ratio. $Pd(dba)_2$ (0.03 mmol, 17 mg) 2-dicyclohexylphosphino-2',4',6'-triisopropylbiphenyl (XPhos, 0.045 mmol, 22 mg), and sodium tert-butoxide (NaOtBu, 2 mmol, 192 mg) was added and placed under a nitrogen atmosphere. Anhydrous toluene (50 mL) was added, and the reaction mixture was heated with stirring to 110°C. After 48 h, the reaction was cooled to room temperature and solvents were then removed by centrifugation. The remaining solids were washed with chloroform, hot deionized water, and methanol (200 mL each) to remove the catalyst, salt and oligomers, followed by Soxhlet extraction with methanol and chloroform,

respectively for 24 h each. The product was then dried in a vacuum oven to yield corresponding amine networks as powder.

Table S. 16 Input information (UV/Vis absorbance) of CMP-BP-1 (Group 10).

CAS	Solvent	Indicator (UV/Vis absorbance at λ_{max})
67-68-5	DMSO	1.23
68-12-2	DMF	1.09
75-05-08	Acetonitrile	0.14
872-50-4	NMP	1.26
108-32-7	Propylene carbonate	1.08
1330-20-7	Xylene	0.12
108-88-3	Toluene	0.25
64-17-5	Ethanol	0.10
123-91-1	Dioxane	0.13
67-66-3	Chloroform	0.98
67-64-1	Acetone	0.15
75-09-2	Dichloromethane	0.58
141-78-6	Ethyl acetate	0.10
110-54-3	Hexane	0.01
109-99-9	THF	0.79
7732-18-5	Water	0.50

3.6.9. Group 11: CMP-BP-4

A Schlenk tube was charged with tris(4-bromophenyl)amine (0.60 mmol, 289 mg) as core, 4,4'-dibromo-2,2'-bipyridine (DBBP) (0.40 mmol, 125 mg) as co-monomer, and *p*-phenylenediamine (1.5 mmol, 162 mg) as linker to obtain a 6:4:15 ratio. Pd(dba)$_2$ (0.03 mmol, 17 mg) 2-dicyclohexylphosphino-2’,4’,6’-triisopropylbiphenyl (XPhos, 0.045 mmol, 22 mg), and sodium tert-butoxide (NaOtBu, 2 mmol, 192 mg) was added and placed under a nitrogen atmosphere. Anhydrous toluene (50 mL) was added, and the reaction mixture was heated with stirring to 110 °C. After 48 h, the reaction was cooled to room temperature and solvents were then removed by centrifugation. The remaining solids were washed with chloroform, hot deionized water, and methanol (200 mL each) to remove the catalyst, salt and oligomers, followed by Soxhlet extraction with methanol and chloroform, respectively for 24 h each. The product was then dried in a vacuum oven to yield corresponding amine networks as powder.

Table S. 17 Input information (UV/Vis absorbance) of CMP-BP-4 (Group 11).

CAS	Solvent	Indicator (UV/Vis absorbance at λ_{max})
67-68-5	DMSO	1.27
68-12-2	DMF	1.13
75-05-08	Acetonitrile	0.66
872-50-4	NMP	1.26
108-32-7	Propylene carbonate	0.92
1330-20-7	Xylene	0.01
108-88-3	Toluene	0.09
64-17-5	Ethanol	0.27

123-91-1	Dioxane	0.31
67-66-3	Chloroform	1.01
67-64-1	Acetone	0.74
75-09-2	Dichloromethane	0.59
141-78-6	Ethyl acetate	0.27
110-54-3	Hexane	0.09
109-99-9	THF	0.46
7732-18-5	Water	0.06

3.6.10. Group 12: PPAAQ

A Schlenk tube was charged with tris(4-bromophenyl)amine (TBPA) (0.30 mmol, 144 mg) and 2,6-diaminoanthraquinone (DAAQ) (0.45 mmol, 107 mg), $Pd(dba)_2$ (0.03 mmol, 17 mg), 2-dicyclohexylphosphino-2’,4’,6’-triisopropylbiphenyl (XPhos, 0.045 mmol, 22 mg), and sodium tert-butoxide (NaOtBu, 4.20 mmol, 404 mg), and placed under a nitrogen atmosphere. Toluene (30 mL) was added to reaction the reaction mixture and sonicated for 10 min to obtain a homogeneous mixture. The reaction mixture was stirred constantly at room temperature for 2 h before heating with constant stirring to 110 °C for 48 h. The reaction was cooled to room temperature and solvents were removed by vacuum filtration. The remaining solids were washed with chloroform, methanol, ethanol and acetone (200 mL each), and dried at 100 °C for 72 h in a vacuum oven.

Table S. 18 Input information (UV/Vis absorbance) of PPAAQ (Group 12).

CAS	Solvent	Indicator (UV/Vis absorbance at λ_{max})
91-22-5	Quinoline	1.29
872-50-4	NMP	1.37
67-68-5	DMSO	1.36
108-32-7	Propylene carbonate	1.18
123-91-1	Dioxane	1.20
68-12-2	DMF	1.19
616-47-7	1-Methylimidazole	1.33
75-05-08	Acetonitrile	1.12
1330-20-7	Xylene	1.25
64-17-5	Ethanol	1.11
108-88-3	Toluene	1.23
127-19-5	Dimethylacetamide	1.23
67-64-1	Acetone	1.14

3.6.11. Group 13: PBAQ

A Schlenk tube was charged with 1,3,5-tribromobenzene (TBB) (0.30 mmol, 94 mg) and 2,6-diaminoanthraquinone (DAAQ) (0.45 mmol, 107 mg), $Pd(dba)_2$ (0.03 mmol, 17 mg), 2-dicyclohexylphosphino-2’,4’,6’-triisopropylbiphenyl (XPhos, 0.045 mmol, 22 mg), and sodium tert-butoxide (NaOtBu, 4.2 mmol, 403 mg), and placed under a nitrogen atmosphere. Toluene (30 mL) was added to reaction mixture and sonicated for 10 min to obtain a homogeneous mixture. The reaction mixture was kept stirring at room temperature for 2 h before heating with constant stirring to 110 °C for 48 h. Once completed, the reaction was cooled to room temperature and solvents were removed

by vacuum filtration. The remaining solids were washed by chloroform, methanol, ethanol and acetone (200 mL each), and dried at 100 °C for 72 h in a vacuum oven.

Table S. 19 Input information (UV/Vis absorbance) of PBAQ (Group 13).

CAS	Solvent	Indicator (UV/Vis absorbance at λ_{max})
91-22-5	Quinoline	1.13
872-50-4	NMP	1.34
67-68-5	DMSO	1.32
108-32-7	Propylene carbonate	0.98
123-91-1	Dioxane	1.08
68-12-2	DMF	1.21
616-47-7	NMI	1.31
75-05-08	Acetonitrile	0.53
1330-20-7	Xylene	0.16
64-17-5	Ethanol	0.47
108-88-3	Toluene	0.10
127-19-5	Dimethylacetamide	1.20
67-64-1	Acetone	0.91
203-577-9	m-Cresol	1.20

3.6.12. Group 14: PPAHQ

A Schlenk tube was placed in an oil bath and charged with tris(4-bromophenyl)amine (TBPA) (0.30 mmol, 145 mg) and 2,5-diaminohydroquinone dihydrochloride (DAHQ) (0.45 mmol, 96 mg), $Pd(dba)_2$ (0.03 mmol, 17 mg), 2-dicyclohexylphosphino-2',4',6'-triisopropylbiphenyl (XPhos, 0.045 mmol, 22 mg), and sodium tert-butoxide (NaOtBu, 12.60 mmol, 1414 mg), and placed under a nitrogen atmosphere. Toluene (30 mL) was added to the reaction mixture and sonicated for 10 min to obtain a homogeneous mixture. The reaction mixture was kept stirring at room temperature for 2 h before heating with constant stirring to 75 °C for 96 h. Once completed, the reaction was cooled to room temperature and solvents were removed by vacuum filtration. The remaining solids were washed by chloroform, methanol, ethanol, and acetone (200 mL each), and dried at 100 °C for 72 h in a vacuum oven.

Table S. 20 Input information (UV/Vis absorbance) of PPAHQ (Group 14).

CAS	Solvent	Indicator (UV/Vis absorbance at λ_{max})
91-22-5	Quinoline	0.73
872-50-4	NMP	1.08
67-68-5	DMSO	1.19
108-32-7	Propylene carbonate	1.09
123-91-1	Dioxane	1.11
68-12-2	DMF	1.13
616-47-7	NMI	1.15
75-05-08	Acetonitrile	1.09
1330-20-7	Xylene	0.44
64-17-5	Ethanol	1.12
108-88-3	Toluene	0.59

127-19-5	Dimethylacetamide	1.12
67-64-1	Acetone	0.95
203-577-9	m-Cresol	1.16

3.6.13. Group 15: PBHQ

A Schlenk tube was placed into oil bath and charged with 1,3,5-tribromobenzene (DBB) (0.3 mmol, 94 mg) and 2,5-diaminohydroquinone dihydrochloride (DAHQ) (0.45 mmol, 96 mg), $Pd(dba)_2$ (0.03 mmol, 17 mg), 2-dicyclohexylphosphino-2',4',6'-triisopropylbiphenyl (XPhos, 0.045 mmol, 22 mg), and sodium tert-butoxide (NaOtBu, 12.60 mmol, 1414 mg), and placed under a nitrogen atmosphere. Ethanol (30 mL) was added to reaction mixture and sonicated for 10 min to obtain a homogeneous mixture. The reaction mixture was kept stirring at room temperature for 2 h before heating with constant stirring to 75 °C for 96 h. Once completed, the reaction was cooled to room temperature and solvents were removed by vacuum filtration. The remaining solids were washed by chloroform, methanol, ethanol and acetone (200 mL each), and dried at 100 °C for 72 h in a vacuum oven.

Table S. 21 Input information (UV/Vis absorbance) of PBHQ (Group 15).

CAS	Solvent	Indicator (UV/Vis absorbance at λ_{max})
91-22-5	Quinoline	0.51
872-50-4	NMP	1.07
67-68-5	DMSO	1.20
108-32-7	Propylene carbonate	1.09
123-91-1	Dioxane	1.12
68-12-2	DMF	1.12
616-47-7	1-Methylimidazole	1.13
75-05-08	Acetonitrile	1.09
1330-20-7	Xylene	0.02
64-17-5	Ethanol	1.11
108-88-3	Toluene	0.035
127-19-5	Dimethylacetamide	1.12
67-64-1	Acetone	0.81
203-577-9	m-Cresol	1.16

3.6.14. Group 16: NPI-4

A dried round-bottomed flask equipped with mechanical stirring, nitrogen inlet, Dean-Stark trap and a reflux condenser was charged with DMF (50 mL), tris(4-aminophenyl)triazine (TAPT) (0.79 mmol, 280 mg) and salt (0.33, 0.66, and 0.99 mmol, respectively). After 5 min of stirring 1,4,5,8,-naphthalenetetracarboxylic dianhydride (NTCDA) (1.19 mmol, 321 mg) was added and the reaction mixture stirred at room temperature for 30 min. The temperature was raised gradually to 150 °C and held for 72 h in a high temperature oil bath. After cooling to 70 °C, MeOH (50 mL) was added and the precipitate was collected and washed with additional DMF and methanol, water and acetone (3 × 50 mL each). The resulting product was dried at 80 °C under vacuum for 24 h.

Table S. 22 Input information (UV/Vis absorbance) of NPI-4 (Group 16).

CAS	Solvent	Indicator (UV/Vis absorbance at λ_{max})
67-68-5	DMSO	1.26

68-12-2	DMF	1.23
75-05-08	ACN	0.94
872-50-4	NMP	1.20
616-47-7	NMI	1.19
108-32-7	Propylene carbonate	0.98
91-22-5	Quinoline	0.95
203-577-9	m-Cresol	1.25
1330-20-7	Xylene	0.40
108-88-3	Toluene	0.19
64-17-5	Ethanol	0.91
123-91-1	Dioxane	1.11
108-67-8	Mesitylene	0.41

3.6.15. Group 17: PTPA-3

A Schlenk tube was charged with tris(4-bromophenyl)amine (0.50 mmol, 241 mg), 4,4′-diaminostilbene dihydrochloride (0.75 mmol, 212 mg), bis(dibenzylideneacetone)palladium(0) (Pd(dba)2, 0.03 mmol, 17 mg), 2-dicyclohexylphosphino-2′,4′,6′-triisopropylbiphenyl (XPhos, 0.05 mmol, 22 mg), sodium tert-butoxide (NaOtBu, 4.20 mmol, 404 mg) and sodium fluoride (NaF, 0.50 mmol, 21 mg). Toluene (50 mL) was added under a N_2 atmosphere. The reaction mixture was stirred for 1 h before heating to 110 °C with stirring and kept under an inert atmosphere for 24 h. After cooling, the resulting product was collected by centrifugation and dried at 70 °C under vacuum before being purified with water (12 h), methanol (12 h), ethanol (12 h) and chloroform (12 h) in a Soxhlet extraction set-up to remove residual catalyst, impurities and any oligomers. The insoluble polymeric materials were then dried under vacuum at 50 °C for 24 h.

Table S. 23 Input information (UV/Vis absorbance) of PTPA-3 (Group 17).

CAS	Solvent	Indicator (UV/Vis absorbance at λ_{max})
123-91-1	1,4-Dioxane	0.72
872-50-4	1-Methyl-2-pyrrolidone	2.57
616-47-7	1-Methylimidazole	3.06
75-05-08	Acetonitrile	0.6
110-82-7	Cyclohexane	0.48
67-68-5	Dimethyl sulfoxide	0.85
68-12-2	Dimethylformamide	3.01
64-17-5	Ethanol	3.21
56-81-5	Glycerol	0.23
7732-18-5	Water	0.00023
67-56-1	Methanol	0.75
127-19-5	N,N-Dimethylacetamide	2.45
108-32-7	Propylene carbonate	1.55
109-99-9	Tetrahydrofuran	1.04
108-88-3	Toluene	0.77
1330-20-7	Xylene	0.51

3.7. Solvent effect on yield for Group 7 to 11 and Group 17 – Screening results

Table S. 24 Yield data for screening solvents with polymers in Group 7, 8, 9, 10, 11, and 17, shown with respect to the Hansen distance calculated by MLoc.

POP name	Group	Solvent	R/ MPa$^{1/2}$	Yield/%
CMP-BP-1	10	DMSO	4.9	0.5
CMP-BP-1	10	PC	8.8	15.8
CMP-BP-1	10	Ethanol	11.8	6.0
CMP-BP-1	10	Toluene	12.4	7.1
CMP-BP-4	11	DMSO	5.1	0.5
CMP-BP-4	11	PC	8.2	23.5
CMP-BP-4	11	Ethanol	12.8	4.2
CMP-BP-4	11	Toluene	12.2	6.4
CMP-TP-1	8	DMSO	3.7	0.9
CMP-TP-1	8	PC	8.1	19.2
CMP-TP-1	8	Ethanol	12.1	6.4
CMP-TP-1	8	Toluene	13.6	7.3
CMP-TP-4	9	DMSO	3.9	0.9
CMP-TP-4	9	PC	8.5	39.0
CMP-TP-4	9	Ethanol	11.7	5.7
CMP-TP-4	9	Toluene	13.6	5.9
PTPA163	7	THF	0.01	5.4
PTPA163	7	1, 4-Dioxane	5.9	4.4
PTPA163	7	Toluene	7.8	36.8
PTPA-3	17	DMF	1.9	2.0
PTPA-3	17	THF	7.6	31.0
PTPA-3	17	Toluene	16.4	5.0

4. Database structure of the HSP-POP database

4.1. The key information included in the HSP-POP database

Table S. 25 An overview of key information included in the HSP-POP database.

Tab name	Introduction
Overview	An overview of current POPs collected in this database. Information includes building block components, stoichiometric ratios, reaction mechanism, HSPs predicted by *MLoc*, best solvents proposed according to the best matching of Hansen distance, best solvents that match in chemical compatibility with other reagents used in corresponding reactions, contributors and references. See Table S. 26 for details.
Building_blocks	Detailed information for building blocks used in this database. Information includes the full name, 2D structural descriptors, abbreviations, and HSPs calculated using the Stefanis group contribution method. [a] See Table S. 27 for details.

MLoc_ip_data	The input UV/Vis data for the *MLoc* workflow used for each POP. See Table S. 28 for details.
MLoc_best_solv	Summary of best solvents predicted for each POP according to both the Hansen distance and chemical compatibility with other reagents used for corresponding reaction mechanism. See Table S. 29 for details.
MLoc_full_detail	Full calculation details for each POP. Information includes HSPs and Hildebrand solubility parameter of each test solvent, differences in HSPs, Hildebrand solubility parameter and Hansen distance between each POP with each solvent candidate. See Table S. 30 for details.
reaction_details	Full reaction details used for the synthesis of each POP. Information includes all reported reaction variables in each system, such as solvents, additives, catalysts, catalyst loadings, stoichiometric ratios, reaction time, temperature, reagents used for workup and purification, etc., and available reported data for each reaction performance. See Table S. 31 for details.

[a]Note that the Stefanis group contribution is included here because of appropriate data availability. While alternative and potentially more advanced group contribution methods, such as the GC+ method,[60] have been proposed, they do not cover HSP data for all fragments involved in the building blocks of POPs. However, we encourage the community to explore advanced methods for future investigations of model performance.

The structure of each tab of the HSP-POP database is introduced in detail in this section.

4.2. The "Overview" tab

This tab gives an overview of all POPs involved in this database and corresponding HSPs predicted by the *MLoc* workflow.

Table S. 26 Introduction of the "Overview" tab of the HSP-POP database.

Column	Introduction
ID	The unique ID (primary key) of corresponding POP in this database.
Name	The name of each POP.
Block_#	The abbreviation name of the building block.
Ratio	The stoichiometric ratios between building blocks involved in each POP.
Mechanism	The reaction mechanism for the synthesis of each POP.
MLoc_D	The dispersion HSP predicted by *MLoc*.
MLoc_P	The dipolar HSP predicted by *MLoc*.
MLoc_H	The hydrogen-bonding HSP predicted by *MLoc*.
MLoc_BestSolvent	The solvent with the minimal Hansen distance from *MLoc*-predicted HSPs.
MLoc_Safe_rxn_solvent_if_different	If the best solvent with minimal Hansen distance can lead to chemical compatibility problems with other reagents used in corresponding reactions, this column is the next best solvent that is safe to carry out such reactions.
Contributor	The researcher that contributed to the synthesis and solvent-dependent UV/Vis data measurement.
Alias	Other names of the same POP that could be used in other sources, for example, the author's computing record, experimental report, etc.

4.3. The “Building_blocks” tab

This tab stores the information for building block structures.

Table S. 27 Introduction of the “Building_blocks” tab of the HSP-POP database.

Column	Introduction
Full Name	The full name of corresponding building block.
SMILES	The SMILES strings of corresponding building block.
Abb	The abbreviation for this building block used across this database.
GC_D	The dispersion HSP calculated via the Stefanis group contribution method.[a]
GC_P	The dipolar HSP calculated via the Stefanis group contribution method. [a]
GC_H	The hydrogen-bonding HSP calculated via the Stefanis group contribution method. [a]

[a] Refer to footnote in Table S. 25 for discussion.

4.4. The “MLoc_ip_data” tab

This tab stores the input UV/Vis data used for the *MLoc* workflow to calculate the HSPs of each POP.

Table S. 28 Introduction of the “MLoc_ip_data” tab of the HSP-POP database.

Column	Introduction
Group #	Information below corresponds to POP ID # in the overview tab.
CAS	The CAS No. of each test solvent.
Solvent	The test solvent name.
Indicator	The UV/Vis absorbance of polymer supernatant in corresponding solvents.

4.5. The “MLoc_best_solv” tab

This tab summarizes the best solvent predicted for each POP according to the Hansen distance and chemical compatibility with corresponding reaction mechanism.

Table S. 29 Introduction of the “MLoc_best_solv” tab of the HSP-POP database.

Column	Introduction
ID	The unique ID (primary key) of corresponding POP in this database.
Name	The name of each POP.
Best solvent	The solvent with minimal Hansen distance from the target material. If labelled with *, the solvent is regarded incompatible with other reagents used with the same reaction conditions. The next best solvent that is not expected with compatibility problems is provided in the bracket.
R	The Hansen distance of the proposed best solvent with the target POP.

4.6. The “MLoc_full_detail” tab

This tab summarizes the full calculation details for each POP, including HSPs and Hildebrand solubility parameter of each test solvent, differences in HSPs, Hildebrand solubility parameter and Hansen distance between each POP with each solvent candidate.

Table S. 30 Introduction of the "MLoc_best_solv" tab of the HSP-POP database.

Column	Introduction
No.	The index of solvent tested for the HSPs prediction of target POP.
Name	The test solvent name or the target POP's name, together with the best solvent index based on the minimal Hansen distance.
D	The dispersion HSP of target POP or solvents.
P	The dipolar HSP of target POP or solvents.
H	The hydrogen-bonding HSP of target POP or solvents.
T	The Hildebrand solubility parameter of target POP or solvents.
dD	The difference in dispersion HSP between each solvent and POP.
dP	The difference in dipolar HSP between each solvent and POP.
dH	The difference in hydrogen-bonding HSP between each solvent and POP.
dT	The difference in the Hildebrand solubility parameter between each solvent and POP.
R	The Hansen distance between each solvent and POP.

4.7. The “reaction_details” tab

This tab summarizes the reaction details reported by the initial contributor of each POP and available data for reaction performance. (If any data is missing, the corresponding cell is filled as “-1”.)

Table S. 31 Introduction of the “MLoc_best_solv” tab of the HSP-POP database.

Column	Introduction
ID	The unique ID (primary key) of corresponding POP in this database.
SubGroup	The subgroup ID for the same building block combinations, but with varied other reaction conditions or purification conditions.
Name	The name of each POP.
Block_#	The abbreviation name of the building block.
Block_#_amount/mmol	The amount of the building block (unit: mmol).
Ratio	The stoichiometric ratios between building blocks involved in each POP.
Mechanism	The reaction mechanism for the synthesis of each POP.
Catalyst	The catalyst used for corresponding synthesis.
Catalyst_amount/mmol	The catalyst loadings used for corresponding reaction (unit: mmol).
Ligand	The ligand used for corresponding catalytic reaction.
Ligand_amount/mmol	The amount of ligand used for corresponding catalytic reaction (unit: mmol).
Base	The base used for corresponding catalytic reaction.
Base_amount/mmol	The amount of base used for corresponding catalytic reaction.
Inert	The environment of corresponding reaction to carry out. If “Y”, the reaction is done with inert environment.
Solvent_for_synthesis	The solvent used for the synthesis.
Solvent_amount/mL	The amount of solvent used for the synthesis (unit: mL).
Reaction_temp/dC	The temperature of corresponding reactions (unit: degree Celsius).
Reaction_time/h	The reaction time (unit: hour).
Solvent_for_precipitate	If applicable, the solvent used after the reaction to support the precipitation of the polymer products.
Solv_prep_amount/mL	If applicable, the amount of solvent used for precipitating the polymers after the reaction has been completed.
Solvent_for_wash_#	If applicable, the #th solvent used for washing the crude product after filtration from the reaction mixtures.
Solv_wash_#_amount/mL	If applicable, the amount of the #th solvent used for washing the crude product after filtration from the reaction mixtures (unit: mL).
Solvent_for_soxhlet_#	If applicable, the #th solvent used for the Soxhlet extraction of polymer crude products. (Note that by default the Soxhlet extraction is carried out at the boiling point of corresponding solvents.)
Sox_time_#/h	If applicable, the time for the #th solvent used for the Soxhlet extraction of polymer crude products (unit: hour).
Yield	The reported yield of purified polymer products.

5. Benchmarking and comparison with reported methods

During the development of this work, we have been seeking to benchmark the prediction with a standard material. However, when reviewing published HSPs data measured using existing models and methods, we found a number of challenges to determine "true" reported HSP values. Key problems we found in published HSPs of polymers include a common confusion between Hildebrand and Hansen solubility parameters, some degree of unawareness of Hansen distance or its use with wrong coefficients, clear biases in the selection of test solvents, ambiguity when determining a categorical solubility score, and oversimplification by optimizing the three-dimensional parameters in three individual one-dimensional spaces. These issues motivated the development of this theoretical framework.

We have applied the *MLoc* algorithm to predict a more standard, non-POP material, graphene, with its successful application to fine-tuning graphene micro domains.[61] The *MLoc*-predicted HSPs of graphene have been compared to reported HSPs in Ref. A[62] below (Table S. 32).

HSPs of graphene in Ref. A adopted a conventional approach, by plotting the solubility score of a number of test solvents on each dimension of Hansen space, followed by taking the solubility average of each HSP. This is conceptually similar to the previous method adopted in the BXJ approach as discussed in the introduction.

The prediction of δ_T (differed by 1.8 MPa$^{1/2}$), as a simpler, 1-dimensional, total solubility parameter, provides a sanity check, confirming that the total predicted HSPs of *MLoc* are acceptably accurate compared to previous measurements.

The prediction of δ_P (differed by 0.6 MPa$^{1/2}$) is also a good match between two methods.

δ_D gives a fair match (differed by 2.2 MPa$^{1/2}$). This is due to its smaller range when compared to the other partial HSPs, due to its smaller range when compared to the other partial HSPs. The difference in δ_D has been explained in Section 1.8 in that directly taking a weighted average of HSPs of all test solvents risks the mis-scaling of the dispersion dimension because of the difference between Hansen distance and Euclidean distance.

δ_H gives a large discrepancy (6.3 MPa$^{1/2}$). This is due to the solvent selection bias in the reported standard. In Ref. A, only good solvents have been used to obtain the solubility scores. Even though 40 solvents were tested, only 3 solvents were protic, causing a significant bias of δ_H towards a lower value. This problem has been addressed in *MLoc* by providing a diverse distribution of test solvents in the 3D Hansen space.

The successful matching of predicted δ_P and δ_T, on the other hand, supports the validity and efficiency of *MLoc*. For δ_P, where solvent selection covered a comparable regime between two methods, *MLoc* only requires 13 solvents, where 40 were used previously.

Table S. 32 Comparison between MLoc-predicted HSPs and "standard" HSPs of graphene.

Method	δ_D/ MPa$^{1/2}$	δ_P/ MPa$^{1/2}$	δ_H/ MPa$^{1/2}$	δ_T/ MPa$^{1/2}$
MLoc	16.2	9.9	14.0	23.5
Ref A[62]	18.0	9.3	7.7	21.7

The input information for graphene and prediction details are provided in Table S. 33.

Table S. 33 Input information (UV/Vis absorbance) and MLoc-predicted HSPs of graphene.

Compound	Abs.	δ_D/ MPa$^{1/2}$	δ_P/ MPa$^{1/2}$	δ_H/ MPa$^{1/2}$	δ_T/ MPa$^{1/2}$	R/ MPa$^{1/2}$
**Graphene**[M]	N/A	16.18	9.87	13.99	23.55	0
Xylene	0.16	17.60	1.00	3.10	17.90	14.33
Hexane	0.18	14.90	0.00	0.00	14.90	17.31
Ethyl acetate	2.30	15.80	5.30	7.20	18.15	8.22
Ethanol	5.37	15.80	8.80	19.40	26.52	5.57
Toluene	0.20	18.00	1.40	2.00	18.16	15.12
PC	4.38	20.00	18.00	4.10	27.22	14.91
DMF	0.63	17.40	13.70	11.30	24.86	5.28
Acetone	1.03	15.50	10.40	7.00	19.94	7.13
Isopropanol	3.17	15.80	6.10	16.40	23.58	4.54
THF	1.53	16.80	5.70	8.00	19.46	7.40
Acetonitrile	3.20	15.30	18.00	6.10	24.40	11.46
Water	0.55	15.50	16.00	42.30	47.81	29.00
Methanol	2.85	15.10	12.30	22.30	29.61	8.92

References

1 R. W. Taft, J. L. M. Abboud, M. J. Kamlet and M. H. Abraham, *J. Solution Chem.*, 1985, **14**, 153–186.

2 E. Stefanis and C. Panayiotou, *Int. J. Thermophys.*, 2008, **29**, 568–585.

3 A. Chandrasekaran, C. Kim, S. Venkatram and R. Ramprasad, *Macromolecules*, 2020, **53**, 4764–4769.

4 M. Chastrette, M. Rajzmann, M. Chanon and K. F. Purcell, *J. Am. Chem. Soc.*, 1985, **107**, 1–11.

5 R. Carlson and J. E. Carlson, *Org. Process Res. Dev.*, 2005, **9**, 680–689.

6 A. R. Katritzky, D. C. Fara, M. Kuanar, E. Hur and M. Karelson, *J. Phys. Chem. A*, 2005, **109**, 10323–10341.

7 D. Mathieu, *ACS Omega*, 2018, **3**, 17049–17056.

8 P. M. Murray, F. Bellany, L. Benhamou, et al., *Org. Biomol. Chem.*, 2016, **14**, 2373–2384.

9 Z. Ye and D. Ouyang, *J. Cheminform.*, 2021, **13**, 1–13.

10 H. Lim and Y. J. Jung, *Chem. Sci.*, 2019, **10**, 8306–8315.

11 G. Ignacz and G. Szekely, *J. Memb. Sci.*, 2022, **646**, 1–10.

12 M. C. Sorkun, J. M. V. A. Koelman and S. Er, *iScience*, 2021, **24**, 1–11.

13 B. Sanchez-Lengeling, L. M. Roch, J. D. Perea, et al., *Adv. Theory Simulations*, 2019, **2**, 1–10.

14 A. F. M. Barton, *Chem. Rev.*, 1975, **75**, 731–753.

15 M. J. Kamlet, J. L. Abboud and R. W. Taft, *J. Am. Chem. Soc.*, 1977, **99**, 6027–6038.

16 R. W. Taft and M. J. Kamlet, *J. Am. Chem. Soc.*, 1976, **98**, 2886–2894.

17 M. J. Kamlet, J. L. M. Abboud and R. W. Taft, in *Progress in Physical Organic Chemistry*, ed. R. W. Taft, 1st edn., 1981, pp. 485–630.

18 R. W. Taft, J.-L. M. Abboud and M. J. Kamlet, *J. Am. Chem. Soc.*, 1981, **103**, 1080–1086.

19 R. W. Taft and M. J. Kamlet, *J. Chem. Soc., Perkin Trans. 2*, 1979, 1723–1729.

20 M. J. Kamlet, J. L. M. Abboud, M. H. Abraham and R. W. Taft, *J. Org. Chem.*, 1983, **48**, 2877–2887.

21 C. M. Hansen, in *Paint and Coating Testing Manual 15th Edition of the Gardner-Sward Handbook*, ed. J. V. Koleske, ASTM International, West Conshohocken, PA, 2012, pp. 494–2012.

22 F.-M. Raoult, *J. Phys. Theor. Appl.*, 1889, **8**, 5–20.

23 J. H. Hildebrand, *Chem. Rev.*, 1949, **44**, 37–45.

24 J. Chen, W. Yan, E. J. Townsend, et al., *Angew. Chemie - Int. Ed.*, 2019, **58**, 11715–11719.

25 C. M. Hansen, *Ind. Eng. Chem. Prod. Res. Dev.*, 1969, **8**, 2–11.

26 R. F. Blanks and J. M. Prausnitz, *Ind. Eng. Chem.*, 1964, **56**, 67.

27 H. Burrell, in *Solvents Theory and Practice*, ed. R. W. Tess, American Chemical Society, Washington, D.C., 1973, vol. 124, pp. 1–10.

28 C. M. Hansen, *The Three Dimensional Solubility Parameter and Solvent Diffusion Coefficient - Their Importance in Surface Coating Formulation*, Danish Technical Press, Copenhagen, 1967.

29 A. Mroz, B. D. Egleston, J. Sherwood, et al., *Chem. Sci.*, 2025.

30 K. D. Shimizu, *Nat. Chem.*, 2013, **5**, 989–990.

31 J. A. Wagoner and N. A. Baker, *Proc. Natl. Acad. Sci. U. S. A.*, 2006, **103**, 8331–8336.

32 J. W. Pitera and W. F. Van Gunsteren, *J. Am. Chem. Soc.*, 2001, **123**, 3163–3164.

33 L. Yang, C. Adam, G. S. Nichol and S. L. Cockroft, *Nat. Chem.*, 2013, **5**, 1006–1010.

34 M. Muruganathan, J. Sun, T. Imamura and H. Mizuta, *Nano Lett.*, 2015, **15**, 8176–8180.

35 F. L. Huyskens, P. L. Huyskens and A. P. Persoons, *J. Chem. Phys.*, 1998, **108**, 8161–8171.

36 M. C. Etter, *J. Phys. Chem.*, 1991, **95**, 4601–4610.

37 G. R. Desiraju, *Acc. Chem. Res.*, 1996, **4842**, 441–449.

38 L. J. Karas, C. H. Wu, R. Das and J. I. C. Wu, *Wiley Interdiscip. Rev. Comput. Mol. Sci.*, 2020, **10**, 1–15.

39 L. Zhang, Q. Zhu, Y. Zhou, et al., *Nat. Commun.*, 2023, **14**, 1–10.

40 C. M. Hansen, *Hansen Solubility Parameters: A User's Handbook*, CRC press, Boca Raton, 2nd edn., 2007.

41 C. Hansen, S. Abbott and H. Yamamoto, Hansen Solubility Parameters in Practice (HSPiP), https://www.hansen-solubility.com/HSPiP/, (accessed 20 January 2025).

42 B. B. Narzary, U. Karatayeva, J. Mintah, M. Villeda-Hernandez and C. F. J. Faul, *Mater. Chem. Front.*, 2023, **7**, 4473–4481.

43 J. S. M. Lee and A. I. Cooper, *Chem. Rev.*, 2020, **120**, 2171–2214.

44 B. B. Narzary, B. C. Baker and C. F. J. Faul, *Adv. Mater.*, 2023, **35**, 1–7.

45 D. F. Swinehart, *J. Chem. Educ.*, 1962, **39**, 333–335.

46 S. Ata, T. Mizuno, A. Nishizawa, et al., *Sci. Rep.*, 2014, **4**, 1–8.

47 J. D. Hunter, *Comput. Sci. \& Eng.*, 2007, **9**, 90–95.

48 T. pandas development team, *Zenodo*, 2020, DOI: 10.5281/zenodo.3509134.

49 M. L. Waskom, *J. Open Source Softw.*, 2021, **6**, 3021.

50 C. R. Harris, K. J. Millman, S. J. van der Walt, et al., *Nature*, 2020, **585**, 357–362.

51 X. Fang, MLoc example solvent candidates, https://github.com/xueannafang/hsp_mloc_v2/blob/main/example_solvents.csv, (accessed 20 January 2025).

52 X. Fang, MLoc input data sheet, https://github.com/xueannafang/hsp_mloc_v2/blob/main/input_mloc_data.csv, (accessed 20 January 2025).

53 S. Kim, J. Chen, T. Cheng, et al., *Nucleic Acids Res.*, 2025, **53**, D1516–D1525.

54 X. Fang, MLoc built-in database, https://github.com/xueannafang/hsp_mloc_v2/blob/main/db.csv, (accessed 20 January 2025).

55 *Anaconda Inc.*, 2020, https://docs.anaconda.com/.

56 U. Karatayeva and C. F. J. Faul, *Chem. Commun.*, 2025.

57 J. P. Olivier, *J. Porous Mater.*, 1995, **2**, 9–17.

58 G. Srinivas, V. Krungleviciute, Z. X. Guo and T. Yildirim, *Energy Environ. Sci.*, 2014, **7**, 335–342.

59 Y. Liao, J. Weber and C. F. J. Faul, *Chem. Commun.*, 2014, **50**, 8002–8005.

60 A. S. Hukkerikar, B. Sarup, A. Ten Kate, et al., *Fluid Phase Equilib.*, 2012, **321**, 25–43.

61 S. He, B. Demir, P. Bouzy, et al., *ACS Appl. Mater. Interfaces*, 2024, **16**, 27694–27704.

62 Y. Hernandez, M. Lotya, D. Rickard, S. D. Bergin and J. N. Coleman, *Langmuir*, 2010, **26**, 3208–3213.
